# Characterizing RNA 3D structural features from DMS reactivity

**DOI:** 10.1093/nar/gkag672

**Published:** 2026-07-17

**Authors:** D H Sanduni Deenalattha, Chris P Jurich, Bret Lange, Darren Armstrong, Kaitlyn Nein, Sakshi Jain, Brandon G Kircher, Joseph D Yesselman

**Affiliations:** Department of Chemistry, University of Nebraska - Lincoln, 639 North 12th St, Lincoln, NE 68588, United States; Department of Chemistry, University of Nebraska - Lincoln, 639 North 12th St, Lincoln, NE 68588, United States; Department of Chemistry, University of Nebraska - Lincoln, 639 North 12th St, Lincoln, NE 68588, United States; Department of Chemistry, University of Nebraska - Lincoln, 639 North 12th St, Lincoln, NE 68588, United States; Department of Chemistry, University of Nebraska - Lincoln, 639 North 12th St, Lincoln, NE 68588, United States; Department of Chemistry, University of Nebraska - Lincoln, 639 North 12th St, Lincoln, NE 68588, United States; Department of Chemistry, University of Nebraska - Lincoln, 639 North 12th St, Lincoln, NE 68588, United States; Department of Chemistry, University of Nebraska - Lincoln, 639 North 12th St, Lincoln, NE 68588, United States

## Abstract

Dimethyl sulfate (DMS) chemical mapping probes RNA structure, where low reactivity is generally interpreted as Watson–Crick (WC) base pairs and high reactivity as unpaired nucleotides. Studies examining DMS reactivity of RNAs with known 3D structures have identified nucleotides that deviate from this interpretation with distinct solvent accessibility and hydrogen bonding patterns. Understanding the frequency of these outliers and their recurring 3D structural features remains incomplete. To address this, we systematically analyzed DMS reactivity across a library of 7500 RNA constructs containing two-way junctions with known 3D structures. DMS reactivity exists on a continuum over four orders of magnitude, with ∼10% overlap between WC and non-WC nucleotides. Non-WC bases with WC-like protection exhibit increased hydrogen bonding and decreased solvent accessibility, whereas reactive WC pairs tend to flank junctions, correlating with weaker base stacking and greater junction dynamics. Reactivity in noncanonical pairs correlates with specific atomic distances, allowing discrimination among base-pair conformations. These underlying reactivity–distance correlations are independently reproduced under *in vivo*-like probing conditions (37°C, 2 min). Using a single reactivity-derived distance constraint in Rosetta Full-Atom Refinement (FARFAR) recovers native A–G base-pair conformations and lowers root-mean-square deviation to high-resolution structures. These results establish that DMS reactivity provides predictive geometric information for RNA 3D modeling.

## Graphical Abstract

**Graphical Abstract:**
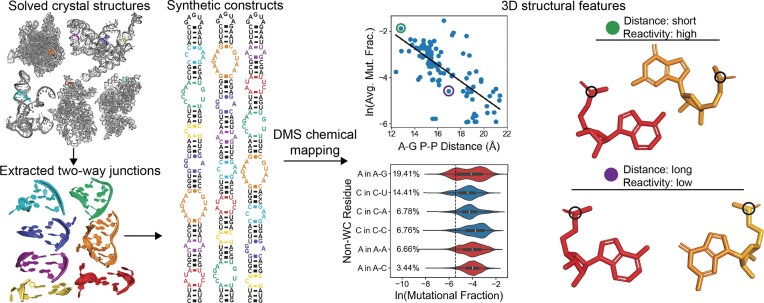

## Introduction

Structured RNAs are pivotal in fundamental biological processes, including protein translation, messenger RNA maturation, and telomere maintenance [1–3]. To perform these functions, many RNAs fold into intricate secondary and tertiary structures that often undergo conformational changes in response to stimuli [4–8]. Elucidating these functions requires knowledge of RNA folding and conformational dynamics.

While high-resolution 3D structures provide valuable atomic-level insights, they capture static snapshots, which may fail to elucidate conformational transitions and their associated energetics crucial for RNA function [9, 10]. Chemical mapping offers an orthogonal and complementary approach to high-resolution structure determination. These approaches employ small-molecule reagents that chemically modify nucleotides based on their local environment, giving insights into dynamic conformation changes and thermodynamics [11–24]. In particular, dimethyl sulfate (DMS) can provide information about RNAs in and out of their cellular contexts, and its use has accelerated due to the ability to read out modifications via next-generation sequencing [24, 25]. These next-generation sequencing approaches can read out DMS methylations at the N1 position of adenine and the N3 position of cytosine, typically leaving nucleotides involved in Watson–Crick (WC) base pairs unmodified. These specific modification patterns have enabled both validation of RNA structures predicted through phylogenetic or thermodynamic approaches and generation of new structural models [24, 26–30]. Furthermore, analysis of RNA sequences that contain multiple DMS modifications in a single read has enabled the computational separation of the data into clusters that reveal distinct RNA secondary structures [31–35].

Recently, we demonstrated that there is a direct relationship between DMS reactivity values and thermodynamics of tertiary contact formation [13]. This ability to provide quantitative information about RNA 3D structure inspired us to confront two critical limitations in current DMS approaches. First, current RNA structure prediction methods use DMS reactivity values to bias folding algorithms through pseudo-energy terms [24, 26–30]. These methods operate at the secondary-structure level: per-residue reactivity is converted to a thermodynamic bonus or penalty that biases whether a nucleotide is assigned to a WC base pair. They are highly effective for secondary-structure determination, but they do not encode information about noncanonical base-pair geometry. Second, over the past decades, there is a wealth of studies that indicate there are exceptions to the general interpretation that low reactivity implies WC pairs and high reactivity are non-WC residues when compared to high-resolution structures. Furthermore, there are cases such as sheared A–G pairs where the A is hyper reactive, displaying reactivity higher than residues that are fully exposed to solvent [12, 20, 36–39]. The structural mechanisms driving these effects stem from variations in solvent accessibility and hydrogen bonding patterns [40–43]. However, the frequency of occurrence, the precise 3D structural features that generate them, and their identification from DMS reactivity data remain unknown.

In this study, we designed a comprehensive RNA library to establish quantitative relationships between DMS reactivity and RNA 3D structure. The library comprised 7500 RNA constructs containing distinct two-way junctions with known 3D structures. DMS reactivity across these constructs forms a continuous distribution with substantial overlap between WC and non-WC nucleotides, and varies systematically with junction asymmetry, base-stacking context, and neighboring base-pair sequence. Reactivity in noncanonical pairs correlates with specific atomic distances, encoding enough information to distinguish among base-pair geometries. To test whether these relationships can constrain RNA 3D modeling, we incorporated a single reactivity-derived phosphate–to-phosphate (P–P) distance constraint into Rosetta Full-Atom Refinement (FARFAR) and demonstrated that it recovers native A–G base-pair geometries that unconstrained modeling fails to find. We then performed an independent validation on 23 A–G-containing motifs whose 3D structures were deposited after our dataset was assembled, where the same single-distance constraint produced comparable improvements in base-pair recovery and root-mean-square deviation (RMSD). Together, these results establish that DMS reactivity encodes predictive 3D geometric information that is complementary to, and operates at a finer level than, the pairing-state bias used by current DMS-guided structure prediction methods.

## Materials and methods

### Extracting isolated two-way junctions from high-resolution RNA structures

We extracted RNA structural motifs from high-resolution experimental structures using the RNA 3D Motif Atlas version 3.162 (http://rna.bgsu.edu/rna3dhub/nrlist) [44]. This database provided nonredundant RNA structures determined by X-ray crystallography and cryo-EM with resolution better than 3.5 Å. Using DSSR (Dissecting the Spatial Structure of RNA) software (version v1.9.9–2020feb06) [45], we identified all structural elements, including n-way junctions, two-way junctions, loops, and helices. At the same time, 3D Structures of Nucleic Acid-Protein complexes (SNAP) software (version lsd 1.1.5) [46] characterized any RNA–protein interactions present in these motifs. We focused on two-way junctions from these elements, applying filters to ensure structural stability when incorporated into designed constructs. Junctions were excluded if they formed more than three hydrogen bonds in total (including interactions with proteins or other RNA motifs) or if they formed more than one hydrogen bond directly involving the nucleotide bases. This process yielded a set of two-way junctions, spanning internal loops, bulges, and motifs like kink turns [47], and sarcin–ricin loops [48], that could maintain their native structural features when isolated. Kink-turns and sarcin–ricin loops are typically considered both 3D and 2D structural motifs; they share the same local architectural rules with internal loops and bulges, namely, a central noncanonical core bracketed by two WC base pairs and are thus categorized within our framework as two-way junctions. This definition also incorporates the flanking base pairs as part of the motif.

All motifs with known 3D structures used in this study are listed in Supplementary Table S1. The PDB of each structure used in this study is included in Fig Share (10.6084/m9.figshare.27880434).

### Selecting symmetric junction sequences that do not have 3D structures

We generated all possible 1 × 1 and 2 × 2 two-way junction sequences, including every combination of flanking base pairs (A–U, U–A, G–C, C–G, G–U, and U–G). From this set, we selected symmetric junctions that did not have experimentally determined 3D structures. To maximize sensitivity to DMS probing, we prioritized sequences containing the highest possible number of adenine and cytosine residues within the non-WC positions, as these nucleotides are most reactive to DMS modification.

### Design of RNA library of 7500 stable hairpin constructs

We designed RNA constructs containing from five to seven two-way junctions arranged in hairpin structures. We define a two-way junction as a secondary structural element comprising unpaired or noncanonically paired nucleotides and flanked by two WC base pairs. Each construct included standardized primer sequences and a central hairpin loop, with non-WC region separated by 5 WC base pairs. Using ViennaRNA [49], we verified that each sequence folded into its intended structure with low ensemble defect scores (≤5). We filtered constructs to ensure lengths were within 10% of the minimum sequence length while not exceeding 150 nucleotides. We required a minimum Hamming distance of 20 between all constructs to maintain sequence diversity. This design process was carried out iteratively using a script that randomly assembled modular RNA motifs with fixed structural elements to form full-length constructs. Each candidate sequence was assessed for correct secondary structure folding, minimal ensemble defect, and a Hamming distance of at least 20 from existing designs. Only sequences that met all criteria were added to the final pool. This loop continued until 7500 unique and structurally validated sequences were generated and compiled into an Agilent oligo pool (sequences provided in Supplementary Table S2).

### Polymerase chain reaction amplification of oligo pool to generate DNA templates

To generate double-stranded DNA (dsDNA) templates for transcription, we amplified the oligo pool using polymerase chain reaction (PCR). The oligo pool was dissolved in 50 μl of 1× IDTE buffer (10 mM Tris-HCl, 0.1 mM EDTA, pH 8.0; Integrated DNA Technologies (IDT), Cat. No. 11-05-01-13). The PCR reaction used forward (TTCTAATACGACTCACTATAGG) and reverse (GTTGTTGTTGTTGTTTCTTT) primers from IDT. Each 50 μl reaction contained 25 μl Q5 High-Fidelity DNA Polymerase (NEB #M0494S), 2 μl oligo pool, 2.5 μl each primer (diluted to 10 μM from 100 μM stock), and 18 μl RNase-free UltraPure water (Thermo Fisher #10 977 015). PCR conditions were: 98°C for 30s, then 18 cycles of 98°C for 10 s, 57°C for 15 s, and 72°C for 15 s, with final extension at 72°C for 5min. Products were separated on 2% agarose gel (150 V, 1 h) and purified using Zymoclean Gel DNA Recovery Kit (Genesee Scientific #11-301C).

### *In vitro* RNA synthesis and purification

RNA was transcribed *in vitro* using a 100 μl reaction containing: 10 μl 10× Transcription Buffer (400 mM Tris–HCl, pH 8.0, 10 mM spermidine, 0.1% Triton X), 5 μl 50 mM Dithiothreitol (DTT), 16 μl 25 mM NTPs, 8 μl 250 mM MgCl_2_, 4 μl T7 polymerase (NEB #M0251S), 24 μl template DNA (adjusted to 0.3 μM), and 33 μl RNase-free water. After 6 h incubation at 37°C, DNA was removed with DNase I and RNA was purified using RNA Clean and Concentrator-5 kit (Genesee Scientific #R1014). Final RNA concentration was measured by nanodrop spectrophotometry and length was verified by 4% denaturing agarose gel electrophoresis (150 V, 1 h).

### Native gel analysis of RNA library

To evaluate whether there were higher order assemblies in the RNA library we performed a native gel. Native 10% polyacrylamide gels were prepared from 2.5 ml of 40% acrylamide/bis-acrylamide (29:1) and 7.5 ml of gel buffer (34 mM Tris, 66 mM HEPES, pH 7.5, 0.1 mM ethylenediaminetetraacetic acid, 3 mM MgCl₂), polymerized with 100 μl of 10% APS and 10 μl of N,N,N',N'-tetramethylethane-1,2-diamine (TEMED). RNA samples were heated in a thermocycler at 90°C for 4 min, snap-cooled to 4°C for 3 min and then folded in the same buffer at room temperature for 30 min. Final RNA concentrations were maintained at 1.5 μM, 0.75 μM, and 0.375 μM before gel loading (Supplementary Fig. 1). We also ran two RNAs with known sizes as controls, both the purine riboswitch and RNase P.

### DMS modification and library preparation for next-generation sequencing

DMS modification was performed on 10 pmol RNA in 5 μl RNase-free water. RNA was denatured (90°C, 4 min), snap-cooled (4°C, 3min), then added to folding solution containing 16.5 μl buffer and 1 μl MgCl_2_ at optimized concentrations. To achieve final concentrations of 0.264 mM sodium cacodylate and 10 mM MgCl_2_, we combined 16.5 μl of 0.4 M sodium cacodylate (pH = 7.16; measured using a pH probe with a 10-fold volume for accuracy, as described in detail by Lange *et al*.) [13] with 1 μl of 250 mM MgCl_2_. RNA was folded at room temperature for 30 min. Meanwhile, the DMS solution was prepared by mixing 15 μl of DMS (Sigma–Aldrich #D186309) with 85 μl of 100% ethanol (Decon Labs #2716). After folding, 2.5 μl DMS solution was added for 6 min, then quenched with 25 μl beta-mercaptoethanol (BME) (Thermo Fisher #125 470 010). For no modification controls, 2.5 μl of H_2_O was added instead of DMS. For our denatured control, we instead folded at 90°C in 7 M urea and probed with DMS for 30 s. Under modified conditions, RNA was folded at 37°C for 30 min and probed with DMS for 2 min at 37°C. The RNA was purified using RNA Clean & Concentrator-5 kit (Genesee Scientific #R1014), eluted in 7 μl RNase-free water, and quantified using Qubit RNA BR Assay Kit (Thermo Fisher #Q10211) using 1 μl sample.

TGIRT-III reverse transcription was used to detect DMS modifications through mutation incorporation. The 12.1 μl reaction contained: 2.4 μl 5× TGIRT buffer (250 mM Tris–HCl, pH 8.3, 375 mM KCl, 15 mM MgCl_2_), 1.2 μl 10 mM dNTPs, 0.6 μl 100 mM DTT, 0.5 μl TGIRT-III enzyme, 6.4 μl modified RNA (diluted to 0.25 μM), and 1 μl barcoded RTB primer (0.285 μM; sequences in Supplementary Table S2). After 2 h incubation at 57°C, RNA was hydrolyzed by adding 5 μl 0.4 M NaOH, heating (90°C, 4min), and snap-cooling (4°C, 3 min). The reaction was neutralized with 2.5 μl quench acid (1.43 M NaCl, 0.57 M HCl, 1.29 M sodium acetate; volume adjusted per batch). The complementary DNA (cDNA) was purified using Oligo Clean and Concentrator Kit (Genesee Scientific #11-380B), adding 30 μl RNase-free water before purification to reach 50 μl total volume. cDNA was eluted in 15 μl RNase-free water.

The cDNA library was amplified using PCR with forward primer AATGATACGGCGACCACCGAGATCTACACTCTTTCCCTACACGACGCTCTTCCG and reverse primer CAAGCAGAAGACGGCATACGAGATCGGTCTCGGCATTCCTGCTGAACCGCTCTTCCGATCTGGGCTTCGGCCC. Each 50 μl reaction contained 25 μl Q5 High-Fidelity DNA Polymerase (NEB #M0494S), 2.5 μl each primer, 2.0 μl purified cDNA, and 18 μl RNase-free water. PCR conditions were: 98°C for 30 s, then 18 cycles of 98°C for 10 s, 62°C for 15 s, and 72°C for 15 s, with final extension at 72°C for 5 min. Correct size bands were excised and purified using Zymoclean Gel DNA Recovery Kit (Genesee Scientific #11-301C). Final library concentration was measured using Qubit 1× dsDNA High Sensitivity Assay Kit (Thermo Fisher #Q33230).

### Generation of DMS reactivity from DMS-MaPseq sequencing data

The sequencing was conducted using Novaseq 6000. The sequencing run was initially demultiplexed using the RTB barcodes inserted during the Reverse Transcription (RT) process, utilizing the novobarcode software (https://www.novocraft.com/documentation/novobarcode/demultiplexing-barcodedindexed-reads-with-novobarcode/).

novobarcode -b rtb_barcodes.fa -f test_R1_001.fastq test_R2_001.fastq

An example for rtb_barcodes.fa would be:

Distance 4

Format 5

RTB021 CCAATGGGTGTA

RTB022 AGCCAAAACTGG

RTB023 GTGTGTTTGCCC

The Distance refers to the variation in base pairs between a barcode and a permissible read when three barcodes are provided. The format specifies that the barcode will be located at the 5′ end of read 1. These demultiplexed fastq files are available on Sequencing Read Archive accession PRJNA1188187.

Processing the demultiplexed fastq files into the mutation fractions was performed by the rna-map software (https://github.com/YesselmanLab/rna_map) [50]. With the following command for each replicate

rna-map -fa < fasta file> -fq1 < R1 fastq file> -fq2 < R2 fastq file> –dot-bracket < csv file>where < fasta file > is the path to the fasta file with all library DNA sequences without T7 promoters. <R1 fastq file > is the path to the R1 fastq file obtained from demultiplexing. <R2 fastq file > is the path to the R2 fastq file obtained from demultiplexing. <csv file > is an optional file that contains the name, RNA sequence and structure in dot bracket notation for each sequence in the library. rna-map will generate a ‘mutation_histos.p’ file that will be used for the next analysis steps.

### Processing DMS reactivity for motif and residue analysis

To ensure high quality data, we filtered our initial dataset of 7500 sequences, removing any sequences with fewer than 2000 reads or signal-to-noise ratios below 4. This filtering step eliminated 17 sequences. We also excluded individual reactivity measurements with z-scores exceeding 3. Here, a z-score represents how many standard deviations a measurement deviates from the mean reactivity at that position across all sequences, allowing us to identify and remove outlier values. This step removed ∼2424 datapoints (∼1% of the total data). These outliers were predominantly found in flanking WC pairs from a small subset of motif sequences (Supplementary Fig. S2 and Supplementary Table S3), suggesting sequence-dependent alternative conformations in these cases.

Each construct sequence was parsed into motifs based on secondary structure and sequence. We defined the first flanking pair as the WC pair directly neighboring noncanonical interactions, which remained constant for motifs derived from 3D structures. The second flanking pair was defined as the next WC pair beyond the first flanking pair, which varied between constructs. To ensure consistent analysis across motifs in all constructs, we standardized motif sequences during the motif parsing step after the DMS experiment. Here, we always placed the strand with greater number of nucleotides first, followed by the shorter strand. For example, a motif originally written as “GG&CAG” (‘&’ is used to separate the two strands) was reordered as “CAG&GG”, as “CAG” is one nucleotide longer than the strand “GG”. If both strands were the same length, they were ordered alphabetically based on their sequence (“GGACG&CUAAC” became “CUAAC&GGACG”). This eliminated the ambiguity in strand orientation and ensured the consistency when representing identical motifs from different constructs, enabling accurate comparisons in analyses.

For each unique motif sequence, we calculated average reactivity values, standard deviations, and coefficients of variation. We then cross-referenced motifs with their corresponding PDB structures to extract detailed structural information. For each nucleotide in each motif, we collected nucleotide residue types (e.g. Flanking-WC, non-WC), sequential position, reactivity data, and pairing information. The residue data were expanded to include neighboring residue types (purine or pyrimidine) and stacking information between nucleotides. After log-transforming residue reactivity data, we mapped each residue to its corresponding PDB file and retrieved additional structural parameters (e.g. B-factors) for residues in the PDB files.

### Logistic regression for predicting WC base pairs from reactivity data

This method employs logistic regression to predict the probability that a base pair is either WC or non-WC based on the natural logarithmic reactivity data of nucleotides. Logistic regression is a binary classification technique that applies a sigmoid function to the input data, producing a probability score between 0 and 1, where values closer to 1 indicate a higher likelihood of the base pair being WC. In this method, the base pair type is first transformed into a binary variable, with WC encoded as 1 and non-WC as 0. The logistic regression model is then trained on the natural logarithmic reactivity data, learning the relationship between reactivity and base pair type. Once trained, the model computes the probability that each base pair is WC.

### Calculating the structural features for nucleotides in motifs that have PDB files

We analyzed the structural parameters of RNA junctions using the 3DNA software package [46]. For each PDB file, the find_pair and analyze commands were executed to generate base-pair parameters.

find_pair test.pdb test.inp

analyze test.inp

From the 3DNA output, we extracted detailed base-pair parameters including classification (WC or non-WC), residue numbers, and geometric measurements (shear, stretch, stagger, buckle, propeller, and opening angles). To assess structural deviations, we calculated RMSDs by aligning base pairs to ideal PDB conformations using the Kabsch algorithm [51]. We then characterized noncanonical base pairs using the Leontis–Westhof (LW) classification system [52], manually comparing each structure to exemplars from the RNA Basepair Catalog [53]. This manual approach avoided misclassification errors we observed with automated methods. Using the Biopython PandasPdb module, we extracted atomic coordinates to calculate pairwise distances between all atoms in residues of interest. Finally, we calculated solvent accessible surface area (SASA) for specific atoms using the freesasa python package [54].

### Reactivity normalization strategies

We applied four normalization strategies to the raw mutation fraction data to account for variation in sequencing depth and experimental conditions. First, each nucleotide’s DMS-induced mutation fraction was divided by its corresponding value in the no-modification control. Second, we normalized each nucleotide by its mutation fraction in the denatured control, which represents the maximal achievable reactivity. Third, for each construct, we calculated the average mutation fraction of all adenine and cytosine residues and divided each nucleotide’s value by this construct-specific average. Finally, we used the two adenines present in the common hairpin included in every construct as an internal reference; the average mutation fraction of these residues was used to normalize all mutation fractions within the dataset.

### Processing of DMS-MaPseq data using the DREEM pipeline

We processed DMS-MaPseq data using the DREEM pipeline to derive mutational profiles indicative of RNA structural ensembles. A locally installed version of DREEM was used for all analyses. Reference sequences were provided in FASTA format, ensuring that each sequence header matched the internal ref_name required by the pipeline. Paired-end sequencing reads were supplied as FASTQ files following the convention

<sample_name>_mate1.fastq and <sample_name>_ mate2.fastq

DREEM was executed from the command line using the syntax:

dreem input_dir output_dir sample_name ref_file ref_name START END –-fastq

An example is shown below:

dreem input output C005V C005V.fasta C005V 1 160 –-fastq

Start and end coordinates were optionally defined to isolate specific domains within larger reference sequences. DREEM generated per-base mutation frequency profiles, which reflect DMS modification rates, and performed clustering to detect alternative RNA structural states. These outputs were used to distinguish coexisting conformations and quantify ensemble heterogeneity across the molecule (Supplementary Table S5).

### Bootstrap estimation of median reactivity ratios

We used nonparametric bootstrapping to quantify the median ratio differences between Flank-WC and non-WC base pairs across various structural contexts. For each data group (“None”, “Random”, “Second Stack”), reactivity values were first subset by base pair type (Flank-WC versus non-WC). We then performed 5000 bootstrap resamples with replacement, independently sampling each distribution to generate paired estimates of the median fold-change in Flank-WC relative to non-WC reactivity. The bootstrap statistic was defined as the ratio of median Flank-WC reactivity to median non-WC reactivity for each iteration.

### Reactivity-derived distance constraints for RNA structure prediction and validation

DMS reactivities were converted to P–P distance estimates using a previously established linear relationship between the natural logarithm of the mutation fraction [ln(r)] and the P–P distance for A–G nucleotide pairs. Using the fitted parameters (m and b, where m and b are the slope and intercept of the linear regression, respectively), the equation was inverted to estimate the interatomic distances (*d*_pred_) directly from experimental reactivity values ${{d}_{{\mathrm{ pred}}}}\ = \ ( {{\mathrm{ln}}( r )\ - \ b} )\ /\ m$.

Predicted interatomic distances were incorporated into the Rosetta constraint file (constraint.cst) for structure modeling. Distance constraints used the AtomPair constraint type, which constrains the distance between two atoms in the modeled structure.

AtomPair P 3 P 6 HARMONIC 13.9 0.5where P are the atom names, and 3 and 6 are the residue numbers for each nucleotide involved. The functional type used is a harmonic function (HARMONIC). 13.9 Å is the predicted P–P distance, and 0.5 Å is the standard deviation.

Next, RNA structure prediction was performed using Rosetta Fragment Assembly of RNA with FARFAR [55]. For each motif, four input files were provided: a FASTA file specifying the RNA sequence, a secondary structure file (secstruct), a constraint file (constraint.cst) containing the predicted distance restraints, and the corresponding native PDB structure for reference. A total of 1000 structural models were generated for each motif using the command below.

rna_denovo.mpi.linuxgccrelease -fasta < fasta file> -secstruct_file < secstruct file> -minimize_rna true -nstruct 1000 -native < native PDB file> -exclude_native_fragments true -constraints:cst_file < constraint file>

After model generation, the resulting silent output file (default.out) was used to extract the models using the command below.

extract_pdbs.mpi.linuxgccrelease -in:file:silent default.out

To determine the optimal parameters for constraint formulation, we systematically evaluated different functional forms and parameter ranges for incorporating predicted distances into Rosetta modeling. Specifically, we compared harmonic and Gaussian constraint functions across a range of parameter values. For the harmonic potential, standard deviation values ranging from 0.5 to 5.0 Å were tested in increments of 0.5 Å. For the Gaussian functional form, the same range of standard deviation values was explored in combination with different constraint weights (1.0, 1.5, and 2.0), which modulate the contribution of the constraint to the overall Rosetta energy function. Across all tested conditions, the harmonic constraint with a standard deviation of 0.5 Å consistently yielded the highest agreement with reference base-pair geometries.

Following structure generation, DSSR was used to annotate base-pair types for A–G pairs in each motif. The analysis was performed using the following command:

x3dna-dssr -i ≤ PDB file> -o ≤ output file>

For each motif, the base-pair types identified in the generated models were compared to those observed in the corresponding native structure, with model accuracy quantified as the fraction of A–G base-pair types correctly predicted, enabling a direct assessment of how well constraint-guided modeling captures native base-pair geometry classified using LW notation. For each selected model, predicted A–G base pairs were extracted and matched to the corresponding nucleotide pairs in the native structure, and a prediction was considered correct only if both the nucleotide pair identity and the assigned base-pair type matched the native annotation; all native A–G pairs were evaluated across all selected models, with missing or mismatched base-pair types treated as incorrect predictions.

To assess the impact of distance constraints, this analysis was conducted separately for models generated with and without constraints. The difference in base-pair recovery between the two conditions was computed for each motif, enabling direct evaluation of the effect of distance constraints on structure prediction accuracy.

## Results

### Designing a massive library to quantitatively relate DMS reactivity to RNA structure

To build a quantitative relationship between RNA structure and DMS reactivity, we developed a systematic approach using RNA elements with known 3D structures. We extracted two-way junctions from the RNA nonredundant database [44]. A two-way junction is a secondary structural element in which two helices are connected by a region containing unpaired or noncanonically paired nucleotides. Throughout this study, we define “WC nucleotides” explicitly as nucleotides engaged solely in canonical WC base pairing interactions (A–U and G–C). Conversely, “non-WC nucleotides” refer to those nucleotides that either participate in noncanonical base-pairing interactions or remain unpaired within the motif. These junctions are ideal for our study because they maintain their structure when isolated from larger RNAs [4, 56–58]. Furthermore, they are fundamental building blocks in functional RNAs, playing critical roles in ligand binding and catalysis [59–61]. We found 178 unique RNA two-way junctions (from 172 3D structures) that were isolatable, i.e. had no more than two hydrogen bonds to nonmotif residues (see the ‘Materials and methods’ section). These two-way junctions encompass a variety of structural elements, including internal loops, bulges, and well-known motifs such as kink turns [47] and sarcin–ricin loops [48] (Supplementary Fig. S3 and Supplementary Table S1) (see the ‘Materials and methods’ section for the classification criteria of these motifs). We supplemented our dataset with 536 1 × 1 and 2 × 2 symmetrical junctions without known 3D structures (Supplementary Table S4). Previous work has shown that these small, symmetric junctions often comprise noncanonical base pairs [62–66]. These additional junctions expand our ability to systematically observe trends among different types of potential noncanonical pairs.

**Figure 1.**
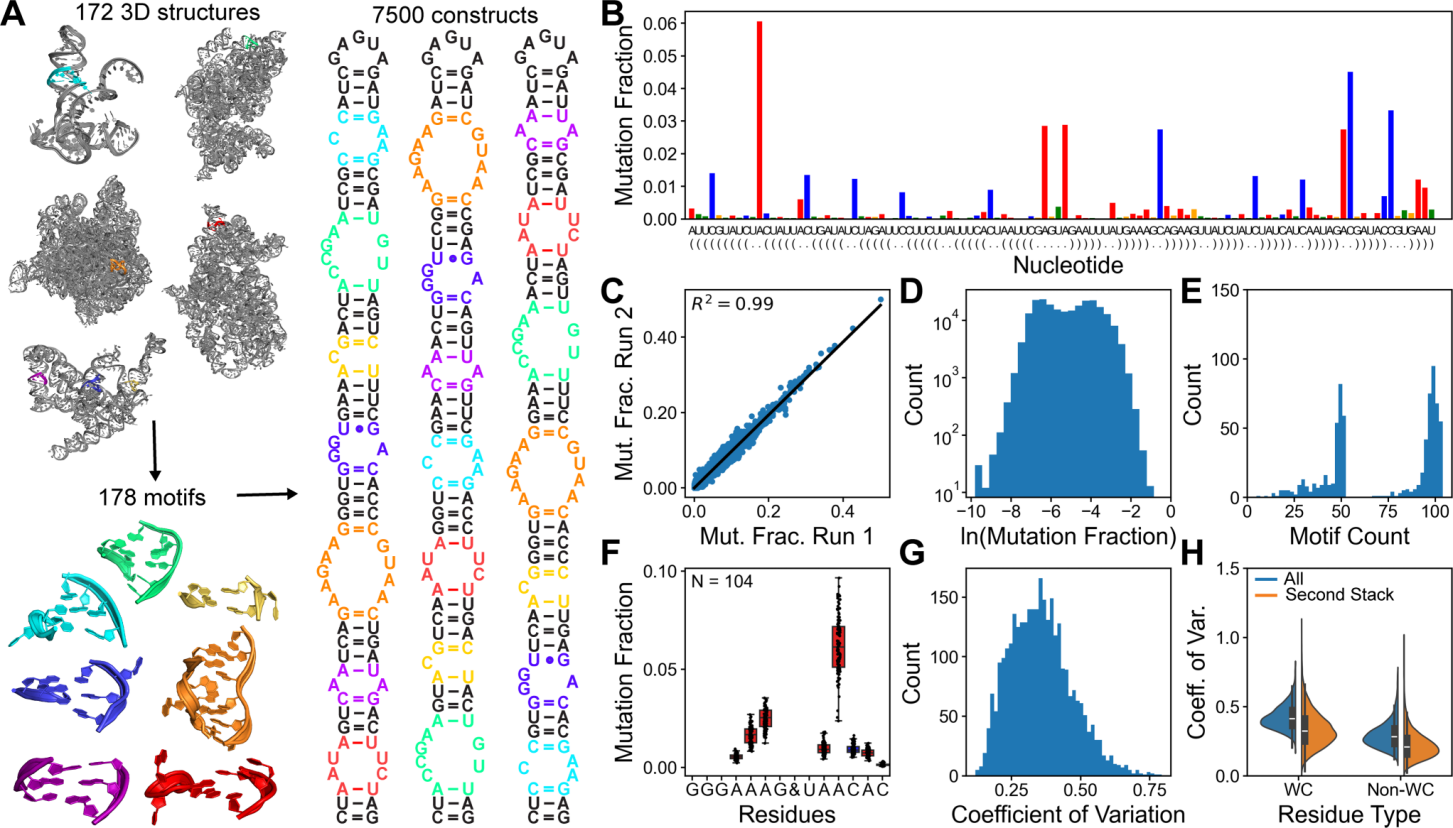
Design and validation of a large-scale RNA library for DMS structure-reactivity analysis. (**A**) Library design strategy: Isolatable RNA motifs were extracted from 3D structures and combined with engineered variants to create stable hairpin constructs containing multiple motifs separated by helices (Supplementary Fig. S30 illustrates the design of the RNA library, which incorporated both known 3D structural motifs and engineered 1 × 1 and 2 × 2 two-way junctions). (**B**) A representative construct shows the secondary structure and DMS reactivity data. Nucleotides are color-coded: adenine (red), cytosine (blue), uracil (green), and guanine (gold). The height of the bars indicates DMS reactivity. (**C**) Reproducibility of DMS measurements across independent experiments. Each point represents a nucleotide’s mutational fraction (R² = 0.99, *n* = 240 000 measurements). (**D**) Distribution of DMS reactivity values shown on a natural logarithmic scale, spanning four orders of magnitude (6.0 × 10⁻⁵ to 0.5). (**E**) Frequency distribution of motif occurrences within the library. (**F**) Example of reactivity consistency: The motif “GGGAAAG&UAACAC” with secondary structure “(…..(&)…..)” shows similar DMS reactivity patterns across multiple sequence contexts, demonstrating measurement reproducibility. (**G**) Measurement variability analysis: Coefficient of variation (CV = standard deviation/mean) for each nucleotide position across all motif instances. (**H**) Impact of structural context: Comparison of CV distributions when nucleotides are grouped by second flanking pair identity versus ungrouped, showing reduced variability with grouping (see Supplementary Fig. S7 to compare to the random grouping of the same size).

We engineered a massive RNA library by incorporating these junctions into 7500 unique RNA constructs. Each construct was designed as a 150-nucleotide sequence containing 5–7 junctions arranged within stable hairpin structures (Fig. 1A). This hairpin architecture was crucial—providing a stable structural scaffold ensured each junction would fold into its intended conformation rather than forming alternative structures (see the ‘Materials and methods’ and Supplementary Results sections). Each junction appears 30 times on average, ranging from 5 to 104 occurrences (Fig. 1E). This redundancy enables the calculation of average DMS reactivity per junction and reveals how local sequence context influences junction reactivity.

### DMS reactivity is highly reproducible and is primarily governed by local sequence and structure

We performed DMS mutational profiling with sequencing (DMS-MaPseq) on our library of 7500 constructs, with a high average read depth of 38 000 reads per sequence (Fig. 1B and Supplementary Fig. S4). Our measurements revealed DMS reactivities spanning four orders of magnitude (6 .0 × 10^−5^ to 0.5). Based on previous research, we employed the natural logarithm of DMS reactivity, which allows for a more intuitive interpretation of the data while preserving the full range of observed reactivities (Fig. 1D) [13]. Replicate experiments showed excellent reproducibility (R² = 0.99) for the 240 000 DMS measurements (Fig. 1C), though the correlation decreased with reactivity values below 0.001 (R² = 0.37; Supplementary Fig. S5). This lower bound corresponds to our no-modification background mutation rate of 0.0014 (Supplementary Fig. S6).

To ensure that the reactivity patterns identified here are not specific to one probing protocol, we also performed DMS-MaPseq on the full 7500-construct library under a short-incubation, higher-temperature protocol (2 min, 37°C; see the ‘Materials and methods’ section) that approximates conditions used for *in vivo* DMS-MaPseq probing. Constructs averaged ∼28 000 reads per sequence. More sequences (684 versus 17 under the standard protocol) failed to meet the quality thresholds (aligned read count >2000 and signal-to-noise ratio >4), reflecting reduced modification of intrinsically low-reactivity WC nucleotides at the shorter incubation time. Per-nucleotide reactivities measured under the two protocols are strongly correlated (R^2^ = 0.79; Supplementary Fig. S7), indicating that the modified protocol recovers the same underlying reactivity signal.

To quantify how consistently each junction behaved across different sequence contexts, we calculated the coefficient of variation (CV)—a standardized measure of variability that divides standard deviation by the mean. A low CV would indicate that a junction’s DMS reactivity remains constant regardless of its position in different constructs. In contrast, a high CV would suggest that surrounding sequences strongly influence its reactivity. We found that the average CV was 0.36 across all nucleotides (Fig. 1H), with WC pairs showing more variability (CV = 0.42) than non-WC residues (CV = 0.30). The elevated CV value for WC pairs is due to the lower DMS reactivity values, which are generally more affected by the number of reads. These differences were not explained by read coverage, as setting higher thresholds had no effect (Supplementary Fig. S8). CV values between 0.2 and 0.3 are usually considered moderate, and above 0.3 is high. These elevated CV values led us to consider other structural effects.

To further investigate the cause of the size of the average DMS reactivity CV, we analyzed the effects of the next WC pair after the flanking pair (the second flanking pair). When we grouped our data based on the second flanking pair identity, the average CV for WC pairs decreased from 0.42 to 0.34, and non-WC residues reduced from 0.30 to 0.22. To ensure these reductions weren’t simply an artifact of dividing our data into smaller groups, we performed a control analysis using random groupings of the same size, which showed significantly smaller CV reductions, 0.37 for WC pairs and 0.27 for non-WC residues (Supplementary Figs S9 and S10). These data indicate that DMS reactivity is reproducible over an extensive range of values and largely depends on local effects, including the sequence of the junction and its neighboring base pairs.

### DMS reactivity values are continuous, and a significant overlap exists between WC and non-WC nucleotides

**Figure 2.**
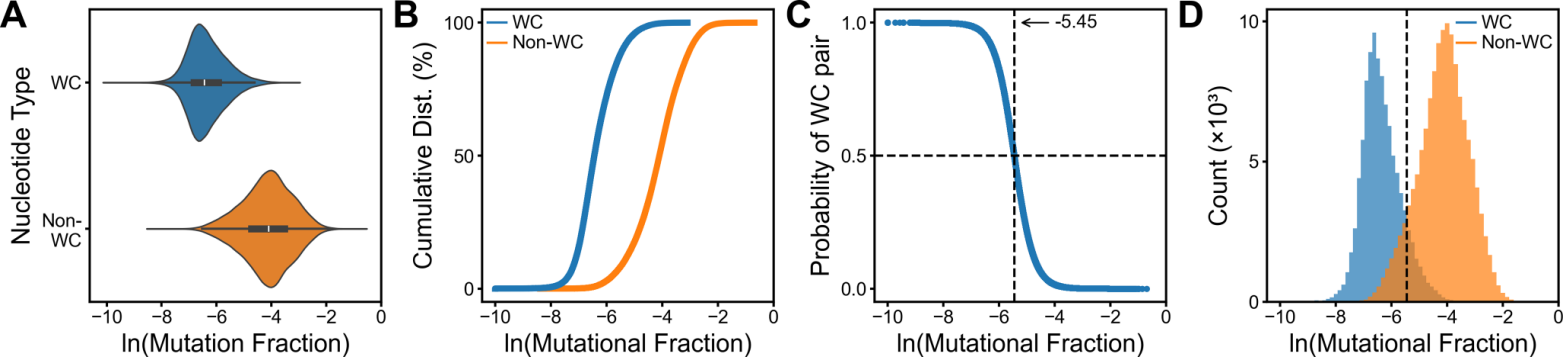
Quantitative analysis of DMS reactivity in flanking WC and non-WC nucleotides. (**A**) Reactivity distributions reveal a significant overlap between nucleotides in flanking WC pairs (blue) and non-WC positions (orange), challenging the simple binary interpretation of DMS data. (**B**) Cumulative reactivity distributions comparing WC paired versus non-WC nucleotides as a function of the natural log of the mutational histogram. (**C**) Logistic regression analysis establishing the probability of WC pairing based on DMS reactivity. The horizontal dashed line marks the 50% probability threshold, corresponding to a natural log mutation fraction of −5.45 (mutation fraction = 0.0043). (**D**) Distribution of nucleotides relative to the 50% probability threshold. While this cutoff optimally separates WC from non-WC nucleotides, significant overlap demonstrates the limitations of using fixed reactivity thresholds for structure prediction.

A key purpose of this study is to systematically investigate the relationship between high-resolution structure features and DMS reactivity. Most RNA structure prediction methods use a pseudo-free-energy term that reduces the likelihood of WC pairing in proportion to reactivity. Based on this assumption, there should be two distinct populations of DMS reactivity values in our dataset, one with low reactivity that is WC pairs, and the other higher for non-WC residues. Comparing the DMS reactivity of flanking WC pairs with non-WC residues does demonstrate there are two distributions however there is about a 10% overlap (Fig. 2A and B) (including nonflanking WC pairs gives similar distributions and analysis, but lacks 3D structural information, see Supplementary Fig. S11).

To quantitatively distinguish WC base pairs from non-WC residues based on DMS reactivity, we applied a logistic regression analysis to determine an optimal reactivity cutoff. This analysis identified a DMS reactivity threshold of 0.0043 (ln = −5.45), corresponding to a 50% probability of a nucleotide being engaged in a WC pair (Fig. 2C and D). Using this cutoff, 8.88% of non-WC residues (12093 of 136115) exhibited low reactivity typical of WC base pairs, whereas 10.71% of WC residues (10860 of 101413) showed unexpectedly high reactivity. Applying the same logistic-regression procedure to the 37°C/2 min data yields a threshold of 0.0024 (ln = −6.05) and a WC/non-WC overlap of 16.2%, which shows a greater overlap, but this is primarily due to the lower reactivity (Supplementary Fig. S12). This reduced reactivity likely arises from the shorter incubation time, which limits the extent of DMS modification of RNA nucleotides. To assess whether normalization could improve the separation between WC and non-WC reactivities, we tested four distinct normalization strategies (see the ‘Materials and methods’ section). None of these approaches significantly reduced the overlap between the two classes, and in some cases, normalization increased it (Supplementary Figs S13 and S14).

### DMS reactivity of flanking WC pairs report on sequence, structure, and dynamics

**Figure 3.**
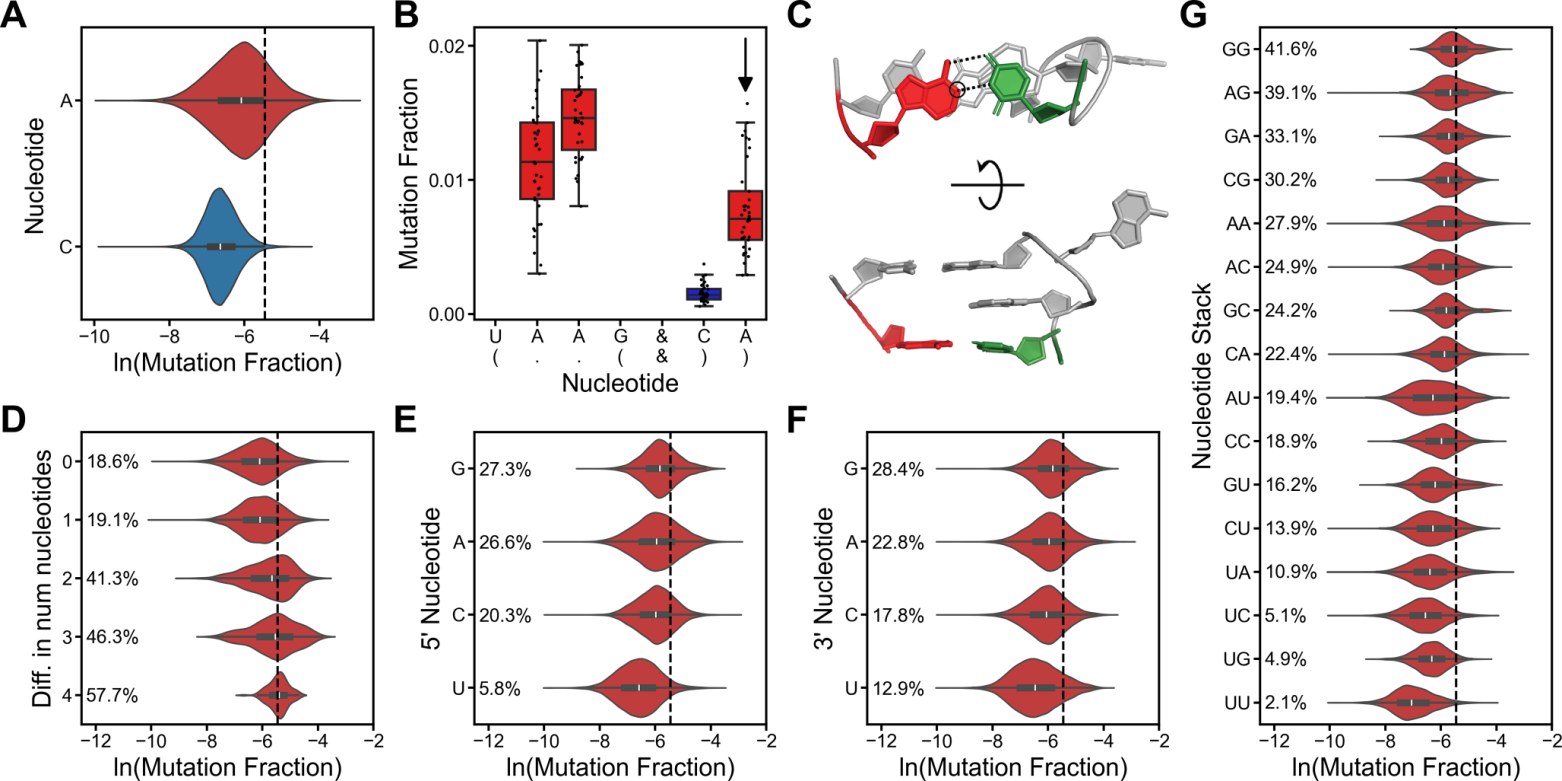
Sequence context and structural features influence WC pair reactivity. (**A**) DMS reactivity distributions comparing adenines in A–U pairs versus cytosines in C–G pairs. The vertical dotted line (natural log of reactivity = −5.45) is the 50% likelihood of being a WC base pair. A–U pairs are frequently more reactive than C–G pairs. (B, C) Example snapshot of a WC pair exhibiting high reactivity. This static structure represents only one conformation and does not capture the full range of possible configurations or dynamic behavior. (**D**) Distributions of the natural log of reactivity for As in A–U flanking pairs as a function of the asymmetry of the non-WC paired residues where 0 is symmetrical, and 4 is four more residues on one side than the other. (**E**) The distribution of the natural log of reactivity as a function of the 5′ residue or the residue that appears right before the A in the A–U pair. (**F**) The distribution of the natural log of reactivity as a function of the 3′ residue or the residue that appears right after the A in the A–U pair. (**G**) The combined influence of flanking sequence context. Two-nucleotide patterns (e.g. “GG” = 5′-GAG-3′) reveal strong neighboring effects, with purine-rich contexts promoting higher reactivity. Similar trends were observed for cytosines (Supplementary Fig. S31).

To characterize the structural features driving DMS reactivity in flanking WC base pairs, we analyzed conformational, and sequence elements associated with high reactivity (reactivity > 0.0043) in these canonical pairs. We first observed a significant difference in the frequency of high reactivity flanking pairs between C–G (1%) and A–U (19%) pairs (Fig. 3A). This substantial difference cannot be attributed to sampling bias, as our dataset contained comparable numbers of A–U and C–G pairs (47652 and 52121, respectively). This significant difference reflects the C–G pair’s greater thermodynamic stability, providing greater protection against DMS modification than A–U pairs. These data indicate that base pair identity plays a role in understanding the difference in DMS reactivity of flanking WC pairs.

To examine whether local structural distortions in WC pairs could account for reactivity differences, we examined each motif’s high-resolution structure. Analysis of 324 high-resolution structures revealed no correlation between DMS reactivity and base pair geometric parameters (shear, stretch, stagger, buckle, propeller, and opening) or overall deviation from ideal geometry (Fig. 3B and C, and Supplementary Fig. S15). Further analysis by specific base pair types (A–U, U–A, G–C, or C–G) revealed no improvement in correlation. These findings indicate that the static, lowest energy conformations of flanking WC pairs, as captured in high-resolution crystal structures, do not provide sufficient information to explain the observed variations in DMS reactivity.

Given the lack of correlation between DMS reactivity and static structural features, we explored the role of RNA dynamics in flanking base pair reactivity. Previous studies suggest symmetric junctions form stable noncanonical pairs, while asymmetric junctions exhibit increased flexibility [62]. We quantified this relationship by analyzing junction asymmetry, which is defined by the difference in residue numbers on each side (0 for symmetric to 4 for highly asymmetric). Our analysis revealed a correlation between junction asymmetry and elevated DMS reactivity in flanking pairs (Fig. 3D). This pattern aligns with known dynamic structures like the 3 × 0 HIV-1 TAR bulge, where the flanking AU pair forms transiently [67].

We also analyzed how local sequence context influences flanking pair reactivity. Examining residues adjacent to flanking pairs revealed sequence-dependent patterns of DMS reactivity (Fig. 3E–G). We found purines at either 5′ or 3′ positions significantly increased the probability of high reactivity in flanking WC pairs. These effects compound flanking pairs with guanines on both sides showed a 40% probability of elevated reactivity compared to only 2% for two uracils. We observed a similar pattern under 37°C/2 min conditions, with a probability of high reactivity for guanines of around 57%, compared to around 8% for uracils (Supplementary Fig. S16E–G). These sequence-dependent reactivity patterns suggest a balance between base stacking and hydrogen bonding in RNA structure. Purine-rich environments favor stacking interactions over hydrogen bonding, increasing flanking pair flexibility and DMS reactivity. This model aligns with multiple high-resolution structures, where stacked purines forgo hydrogen bonding (Supplementary Fig. S17), suggesting a general principle in RNA structural organization.

### Noncanonical base pairs protect against DMS modification through hydrogen bonding and decreased solvent accessibility

**Figure 4.**
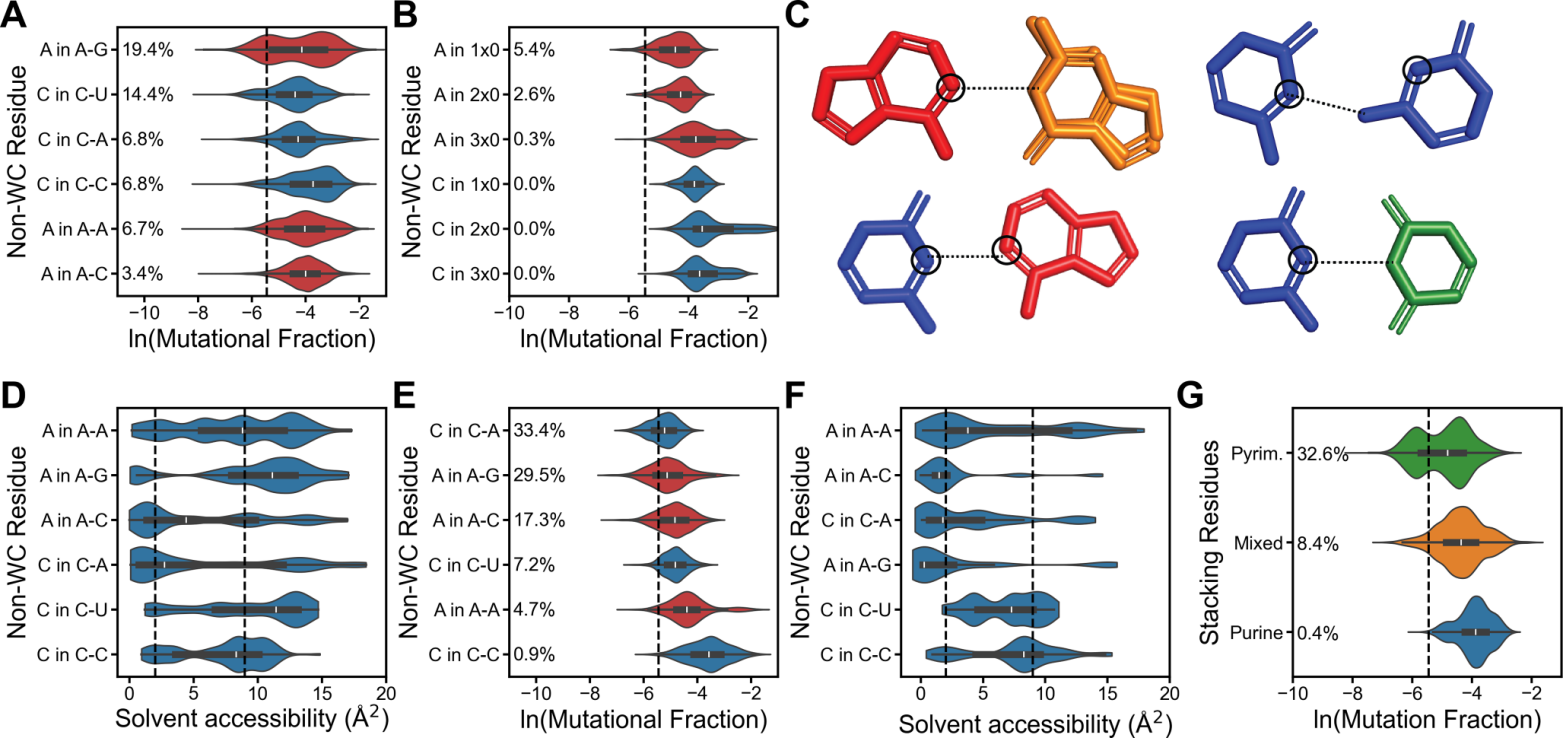
Structural and sequence determinants of low reactivity in noncanonical pairs. (**A**) DMS reactivity distributions for noncanonical pairs with known structures. A vertical dotted line indicates the 50% WC probability threshold (−5.45). Nucleotides left of this line exhibit WC-like protection. (**B**) Reactivity distribution of unpaired nucleotides in bulges, providing baseline comparison for noncanonical pairs. (**C**) Representative examples of conformations of low-reactivity noncanonical pairs: A–G, A-C, C–C, and C–U, colored as A (red), C (blue), G (orange), and U (green). Each illustrates how specific hydrogen bonding patterns can protect DMS modification sites. (**D**) Solvent accessibility of DMS modification sites (adenine N1, cytosine N3) across noncanonical pairs. Vertical lines indicate average accessibility for WC pairs (2 Å^2^ left) and unpaired nucleotides (9 Å^2^, right), demonstrating a correlation between accessibility and reactivity. (**E**) DMS reactivity distributions for noncanonical pairs with known structures in 1 × 1 mismatches. A vertical dotted line indicates the 50% WC probability threshold (−5.45). Nucleotides left of this line exhibit WC-like protection. (**F**) Solvent accessibility analysis focused on 1 × 1 mismatches, revealing distinct patterns of nucleotide protection in symmetric contexts. (**G**) The impact of neighboring sequences on C–U mismatch reactivity shows how local context modulates DMS accessibility in noncanonical pairs.

To characterize structural features causing low DMS reactivity in non-WC nucleotides, we analyzed cases where noncanonical interactions exhibited WC-like protection. To enhance RNA structure prediction accuracy from DMS data, we identified cases where noncanonical interactions could be misinterpreted as WC pairs due to low reactivity. Analysis of nucleotides with known 3D structures revealed that 14.5% of noncanonical pairs showed reactivity below our WC threshold (<0.0043), compared to only 2.0% of unpaired nucleotides. This 7.25-fold difference indicates noncanonical pairing can protect nucleotides from DMS modification similarly to WC pairs (Fig. 4A and B). Under the 37°C/2 min data, the corresponding percentages are 12.4% and 7.3% (Supplementary Fig. S18). Our engineered 1 × 1 and 2 × 2 motifs without known structures confirmed this pattern, where 14.7% and 9% of potential noncanonical pairs, respectively, showed low reactivity. These results indicate that noncanonical interactions frequently generate WC-like protection patterns.

We analyzed reactivity patterns across mismatched pairs (A–A, A–G, A-C, C–C, C–U) using structures with known 3D conformations. Low reactivity frequencies varied by pair type: A–G (19.40%), C in C–U (14.39%), C in C–A (6.78%), C–C (6.75%), A–A (6.65%), and A in A–C (3.44%) (Fig. 4A). This protection stems from hydrogen bonding at DMS modification sites (N1 of adenine, N3 of cytosine). For example, A–G pairs often form *cis* Watson–Crick/Watson–Crick (cWW) arrangements where stable a N1-N1 hydrogen bond provide protection. Similarly, low-reactivity C–A pairs typically show cWW conformations where cytosine’s N3 hydrogen bonds to adenine’s N1. These conformations must involve a protonation of one nucleotide, consistent with previous NMR findings for mismatches in pre-miRNA-31 [68] and other examples reported in the literature. [69–71] (Fig. 4C).

Solvent accessibility plays a crucial role in DMS reactivity. In WC pairs, the N1/N3 positions are shielded from solvent, reducing DMS modification. Analysis of 695 nucleotides from high-resolution structures revealed a moderate correlation (R² = 0.41) between DMS reactivity and solvent accessibility of these modification sites (Supplementary Fig. S19). This relationship extends to noncanonical pairs – those with lower DMS reactivity showed reduced solvent accessibility at N1 and N3 atoms (Fig. 4D). This pattern is most evident in 1 × 1 mismatches, where 33.4% of cytosines in C–A pairs and 29.5% of adenines in A–G pairs demonstrated WC-like reactivity (Fig. 4E). These nucleotides had low solvent accessibility, with 51.4% of C–A pairs and 69.3% of A–G pairs showing values below 2 Å^2^, typical of WC pairs (Fig. 4F and Supplementary Table S6). These findings provide strong evidence that the DMS reactivity of noncanonical pairs can approach that of WC pairs, consistent with the reduced solvent accessibility of the target atoms.

The local stacking environment significantly influences noncanonical pair reactivity. While adenines in A–A, A–G, and A–C pairs showed minimal stacking effects, cytosines in C–A, C–C, and C–U pairs were strongly influenced by their neighboring bases (Supplementary Fig. S20). Pyrimidines flanking these cytosine-containing pairs correlated with reduced reactivity. This effect was particularly dramatic in C–U pairs, 32.63% of cytosines with pyrimidine neighbors showed WC-like reactivity, compared to just 0.36% when flanked by purines (Fig. 4G). This hundred-fold difference likely results from competing structural forces. In pyrimidine-rich environments, cytosine’s weak stacking ability favors hydrogen bond formation with its partner, shielding the N3 position from DMS. Conversely, purine stacking may disrupt hydrogen bonding, exposing the N3 position and increasing DMS reactivity.

### Noncanonical base pairs have distinct reactivity relationships that report 3D structure features

**Figure 5.**
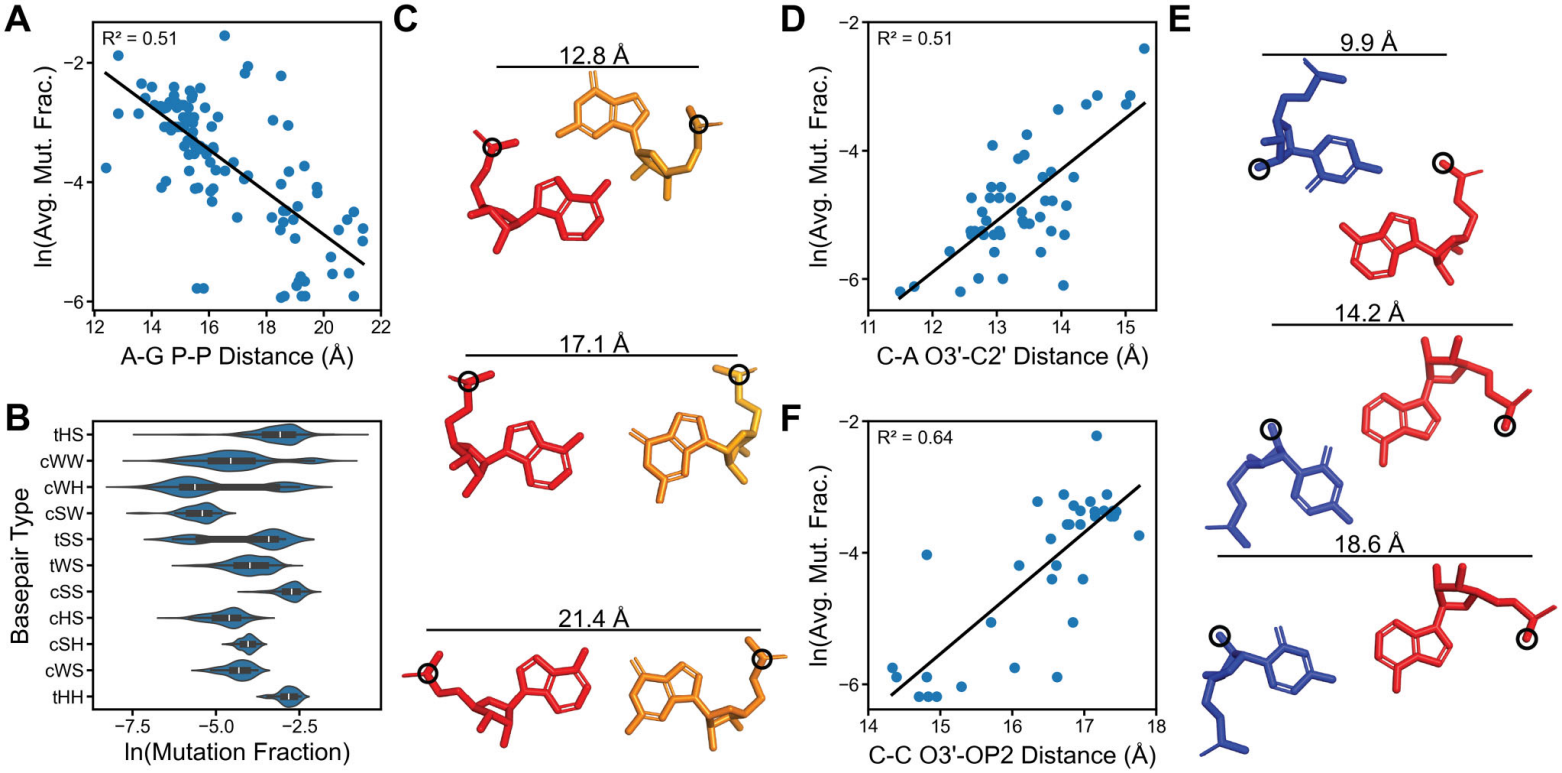
DMS reactivity analysis reveals quantitative relationships with three-dimensional structural parameters in non-canonical base pairs. (**A**) The natural log of adenine reactivity in A–G pairs correlates with phosphate-phosphate distance (R² = 0.51, *n* = 122). (**B**) Distribution of adenine reactivity in A–G pairs by base pair conformation type, showing distinct patterns. (**C**) Representative A–G pairs show short, medium, and long P–P distances. (**D**) Cytosine reactivity in C–A pairs correlates with O3′–C2′ distance (R² = 0.51, *n* = 48). (**E**) Representative C–A pairs show short, medium, and long O2′–OP2 distances. (**F**) The cytosine reactivity in C–C pairs correlates with O3′–OP2 distances.

We tested whether DMS reactivity reports 3D structural features of noncanonical base pairs. We found that A–G, C–A, and C–C reactivity patterns correlate with specific atomic distances, providing insights into base-pair conformations (See Supplementary Fig. S21 for other pairs with weaker correlations). For A–G pairs, we found a correlation (R² = 0.51, *n* = 122) between the phosphate-to-phosphate (P–P) distance and adenine reactivity (Fig. 5A). This correlation resulted from the longest P–P distance in cWW conformations, corresponding to the lowest reactivity values. In contrast, shorter P–P distances were associated with *trans*-Hoogsteen/Sugar (tHS) conformations and the highest reactivity values (Fig. 5B). The same A–G reactivity–P–P distance correlation obtained under the modified probing protocol yields R² = 0.47 on an overlapping set of *n* = 122 pairs, preserving the rank ordering of tHS, cWW, *cis*-Watson–Crick/Hoogsteen (cWH), and *cis*-Sugar/Watson–Crick (cSW) conformations (Supplementary Fig. S22). This pattern suggests that DMS reactivity is sensitive to the overall geometry of the base pair. Further analysis revealed that grouping reactivities by base pair conformation yielded distinct clusters, indicating that the specific interaction type is a primary determinant of reactivity patterns (Fig. 5C). While the P–P distance provided valuable insights, it represents just one of several atomic measurements correlating with base-pairing modes (Supplementary Fig. S23).

C–A pairs showed multiple correlations between atomic distances and reactivity. The strongest correlation (R² = 0.51, *n* = 48) was between cytosine’s O3′ atom and adenine’s C2′ atom (Fig. 5D and Supplementary Fig. S24). Short O3′–C2′ distances corresponded to low cytosine reactivity in tSH and some cWW conformations. Longer distances showed higher reactivity in cWH and tWH arrangements.

C–C pairs showed the strongest correlation (R² = 0.64, *n* = 35) between O3′ and OP2 distances, though with limited samples. Notably, this correlation reveals a structural asymmetry, indicating hydrogen bonding between one cytosine’s O3′ and the other’s OP2 (Fig. 5F and Supplementary Figs S25 and S26). Together, these correlations between DMS reactivity and atomic distances indicate that chemical mapping data encodes 3D structural information of noncanonical pairs.

To assess the predictive power of the observed correlations between chemical reactivity and atomic distances, we extended our analysis to newly available 1 × 1 and 2 × 2 motifs that were not part of the original dataset (Supplementary Table S7). We identified 23 new A–G motif sequences, corresponding to 51 newly solved structures, that match motifs present in the initial dataset. These new structures span the same range of phosphorus–phosphorus (P–P) distances observed previously, and when combined with the original data, the overall correlation improved from R² = 0.51 to R² = 0.62, confirming that reactivity remains a strong predictor of average P–P distance. This represents the most robust case; other base-pair types currently have less extensive data (Supplementary Fig. S27).

For C–A pairs, we identified 13 new sequences corresponding to 39 new structures. Owing to the smaller dataset, these motifs did not cover the full range of distances but generally followed the existing trend, resulting in a modest decrease in correlation from R² = 0.51 to R² = 0.30. Similarly, for C–C pairs, 9 unique sequences with 20 new structures were added. While these spanned a slightly broader range of distances, the overall correlation also decreased from R² = 0.64 to R² = 0.43 (Supplementary Fig. S27). Overall, these data suggest that reactivity can be predictive of atomic distances, but more data is required before the extent of the predictive power is known for each mismatch pair.

### Reactivity-derived distances can predict A–G basepair geometries using 3D modeling

The correlations above suggest reactivity encodes geometric information, but a stronger test is whether they predict specific structural details such as base pairing. We focused on A–G pairs because they are the largest, best-validated subset and because their LW class (cWW, tHS, cWH, cSW) is geometrically diverse, making the prediction nontrivial. Starting only from sequence and the measured DMS reactivity of the adenine, we used the reactivity to predict P–P distance regression (Fig. 5A) to assign a target distance to each A–G pair, then incorporated this as a constraint between the two phosphates in Rosetta FARFAR (see the ‘Materials and methods’ section). For each of the 54 motifs containing one or more A–G pairs, we generated 1000 models with and without the constraint and evaluated the top 100 by Rosetta score on (i) native LW-type recovery and (ii) RMSD to the deposited structure.

**Figure 6.**
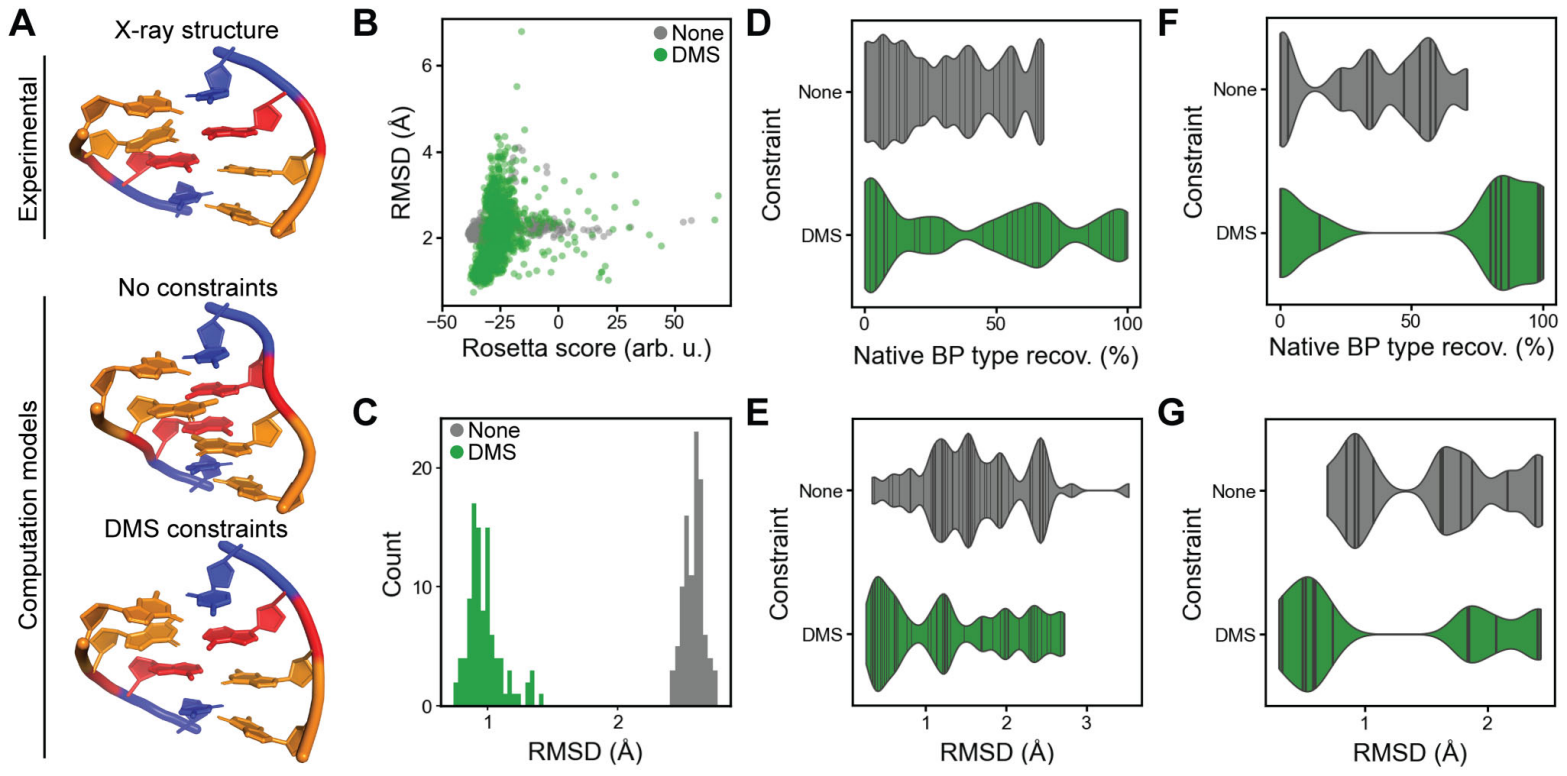
DMS reactivity/distance relationships can predict and improve 3D structure models. (**A**) Representative structures of the GGAC&GGAC 2 × 2 junction. Top: experimental X-ray structure showing two native cWW A–G pairs. Middle: top-scoring FARFAR model without constraints, which converges on the non-native sheared (tHS) configuration. Bottom: top-scoring FARFAR model with a single reactivity-derived P–P distance constraint, recovering the native cWW geometry. (**B**) Rosetta score versus RMSD to the native GGAC&GGAC structure for top 100 models generated without (gray) and with (green) the distance constraint. Without the constraint, sampling is broad and Rosetta score is insensitive to which geometry is adopted; with the constraint, models converge into a tight low-RMSD funnel. (**C**) RMSD distribution of the same top 100 GGAC&GGAC models, showing a high-RMSD distribution without constraint (gray) and a low-RMSD distribution with the constraint (green). (**D**) Per-motif native base-pair-type recovery across the 37 A–G-containing motifs not already correctly modeled by unconstrained FARFAR (motifs with ≥ 80% recovery without constraints excluded), shown as violin plots without (gray) and with (green) the constraint. (**E**) Per-pair RMSD to native across the same 37-motif set, with and without the constraint. (**F, G**) Native base-pair-type recovery (**F**) and per-pair RMSD (**G**) on the held-out set of 23 A–G-containing motifs whose 3D structures were deposited after our dataset was assembled, applying the same single reactivity-derived distance constraint. The constraint produces comparable improvements on this independent set, indicating that predictive performance transfers beyond the structures used to fit the regression.

Seventeen motifs were already modeled correctly by unconstrained FARFAR (≥80% LW-type recovery) and were excluded from further analysis. On the remaining 37 motifs, the single reactivity-derived constraint raised mean native LW-type recovery from 31.3% to 42.2% and brought seven additional motifs above the 80% recovery threshold; performance was never substantially degraded by the constraint (Supplementary Fig. S28). The 2 × 2 junction GGAC&GGAC illustrates the failure mode and how the constraint fixes it. The native structure contains two cWW A–G pairs, but unconstrained FARFAR converges on two sheared (tHS) pairs in 100% of top-scoring models, with Rosetta score insensitive to which geometry is sampled (Fig. 6B). Adding the reactivity-derived P–P distance produces a clean energy funnel toward the native cWW geometry, recovering it in 99.5% of top-scoring models (Fig. 6A and C). Across the 37-motif set, the constraint improves both LW-type recovery and per-pair RMSD (Fig. 6D and E) and notably does so without the constraint specifying which LW class to adopt, the model arrives there from the geometric information alone.

To test whether this predictive capability generalizes beyond the structures used to fit the regression, we performed an independent validation on 23 A–G-containing motifs whose 3D structures were deposited after our original dataset was assembled and were therefore held out from regression fitting (Supplementary Fig. S29). Reactivity values for these motifs came from our original DMS-MaPseq library; the held-out structures provided ground truth. The same single-distance constraint produced comparable improvements in LW-type recovery and RMSD (Fig. 6F and G), confirming that the relationship transfers to structures not used to derive it.

## Discussion

Our systematic analysis of DMS reactivity across 7500 RNA constructs containing known structural motifs and engineered symmetrical junctions reveals several principles relevant to RNA structure determination. These constructs combine known 3D structural motifs with engineered 1 × 1 and 2 × 2 symmetrical junctions, providing data for hundreds of instances of each structural element across different sequence contexts. Results from this library demonstrate that DMS reactivity extends across four orders of magnitude in a continuous distribution. DMS reactivity is broadly reproducible across sequence contexts but is systematically modulated by the identity of neighboring WC pairs in both flanking pairs and noncanonical interactions. Controlling for second flanking pair identity reduced DMS reactivity variability and increased reproducibility. Systematic investigation of second and third flanking pair effects could enhance thermodynamic parameters for RNA structure prediction. Crucially, we show that these reactivity patterns can be used predictively: a single reactivity-derived P–P distance constraint applied to Rosetta FARFAR modeling recovers correct A–G base-pair geometries that unconstrained modeling fails to find, with predictive performance preserved on a held-out set of 23 A–G-containing motifs whose 3D structures were deposited after the dataset was assembled.

Modern RNA structure prediction methods incorporate DMS reactivity through pseudo-energy terms in folding algorithms. These terms favor WC base pairing at positions with low reactivity and disfavor pairing at positions with high reactivity. Prior research identified cases where non-WC residues exhibit low reactivity and WC pairs show high reactivity in specific RNA structures. This study quantified the frequency of DMS reactivity patterns that deviate from the canonical model. The analysis revealed ∼10% of nucleotides diverge from expected patterns, with WC pairs showing higher reactivity and non-WC residues displaying lower reactivity than predicted. However, these outliers should not be interpreted as errors but as potential indicators of 3D structural features.

Multiple structural features influence reactivity patterns that generate DMS outliers. WC pair reactivity depends on base pair composition, with C–G pairs exhibiting five-fold lower reactivity than A–U pairs. Flanking purines and junction asymmetry correlate with increased reactivity. Non-WC residues display reduced reactivity through hydrogen bonding patterns. Overall, 14% of noncanonical residues show WC protection levels, with specific interactions like A–G pairs reaching 20% protection frequency. These noncanonical geometries restrict solvent accessibility to levels comparable to WC pairs. Furthermore, base stacking environments influence reactivity in a position-dependent manner, exemplified by neighboring pyrimidines providing 100-fold greater protection to C–U pairs compared to neighboring purines. This protection derives from competing structural forces—pyrimidines stack poorly, enabling hydrogen bond formation that shields nucleotides from DMS reactivity.

DMS reactivity patterns provide quantitative geometric information for RNA tertiary structure modeling. The P–P distance in A–G pairs correlates with adenine reactivity (R² = 0.51, improving to R² = 0.62 with newly available structures), distinguishing cWW, tHS, cWH and cSW conformations. Additional noncanonical pairs (C–A, C–C) display analogous distance–reactivity relationships. Critically, this geometric information is orthogonal to the per-residue pairing bias used by current DMS-guided structure prediction methods: pseudo-energy formulations determine pairing topology, while reactivity-derived distance constraints determine pairing geometry. Incorporating a single such constraint into Rosetta FARFAR recovers correct A–G geometries in motifs where unconstrained sampling fails, most strikingly the GGAC&GGAC 2 × 2 junction, where two cWW A–G pairs are recovered in 99.5% of top-scoring models with the constraint versus 0% without. This single-distance constraint brings 7 of 37 previously-failing motifs above 80% native LW-type recovery. The relationships transfer to a blind set of 23 A–G motifs whose 3D structures were deposited after our regression was fit, demonstrating that the predictive capability is not an artifact of the training set. Together, these results establish that DMS reactivity contains predictive 3D structural information accessible through geometric, rather than thermodynamic, modeling.

All major reactivity patterns reported in this study, including the WC/non-WC distribution overlap, the flanking-pair sequence dependence, the protection of noncanonical pairs, and the reactivity–distance correlations underlying the modeling results, are reproduced under *in vivo*–like probing conditions (2 min, 37°C), indicating that the conclusions are not specific to a single DMS protocol.

Overall, our findings indicate that DMS chemical mapping data contains more structural information than previously utilized. Future work can build on these relationships between reactivity patterns and structural features to develop more accurate RNA structure prediction methods, particularly for complex structural elements containing noncanonical pairs. Integrating both modalities, pseudo-energy at the secondary-structure level and reactivity-derived distance restraints at the 3D level, within a single pipeline is a natural next step toward DMS-guided de novo RNA 3D modeling, particularly for functional RNAs whose noncanonical pairing geometries are central to activity.

## Supplementary Material

gkag672_Supplemental_Files

## Data Availability

All data, materials, and software used in this study are available. Unprocessed FASTQ files have been deposited to the Sequence Read Archive (SRA) under the accession PRJNA1188187. All other data is available on Fig Share (10.6084/m9.figshare.27880434). All code used in this study is available on GitHub: https://github.com/YesselmanLabPublications/2025_char_3d_struct_features (DOI via Zenodo: 10.5281/zenodo.16884332)
