# Supplementary material for: Characterizing RNA 3D structural features from DMS reactivity
Source: Nucleic Acids Res. 2026 Jul 17;54(14):gkag672. doi: 10.1093/nar/gkag672 (PMC13376268; doi:10.1093/nar/gkag672)
Supplement: gkag672_Supplemental_Files [file gkag672_supplemental_files.zip › 2025_char_3d_struct_features_supplemental_second_revision_clean.docx]

# Supplementary Information for Characterizing RNA 3D structural features from DMS reactivity

D. H. Sanduni Deenalattha^1^, Chris P. Jurich^1^, Bret Lange^1^, Darren Armstrong^1^, Kaitlyn Nein^1^, Sakshi Jain^1^, Brandon G. Kircher^1^ and Joseph D. Yesselman^1^*

^1^Department of Chemistry, University of Nebraska, 639 North 12th St, Lincoln, NE 68588, USA

*Corresponding author: [jyesselm@unl.edu](mailto:jyesselm@unl.edu)

Table of Contents

[Supplementary Information for Characterizing RNA 3D structural features from DMS reactivity 1](#_Toc228227062)

[Supplemental Methods: DREEM clustering of example hairpin constructs. 6](#_Toc228227063)

[Supplemental Figure S1: Native gel of two-way junction library demonstrates no higher-order interactions 7](#_Toc228227064)

[Supplemental Figure S2: DMS reactivity patterns for motifs highlighting outliers 8](#_Toc228227065)

[Supplemental Figure S3: Distribution of two-way junctions 9](#_Toc228227066)

[Supplemental Figure S4: Number of reads per construct 10](#_Toc228227067)

[Supplemental Figure S5: Reproducibility of DMS measurements below 0.001 across independent experiments. 11](#_Toc228227068)

[Supplemental Figure S6: Background mutation rates in the no-modification control are low and uncorrelated with DMS signal 12](#_Toc228227069)

[Supplemental Figure S7: Validation of RNA library for DMS structure-reactivity analysis under modified conditions 13](#_Toc228227070)

[Supplemental Figure S8: Coverage dependence of the CV for Flank-WC and non-WC residues. 15](#_Toc228227071)

[Supplemental Figure S9: Comparison of variability between second flanking pair grouping and random grouping 16](#_Toc228227072)

[Supplemental Figure S10: Bootstrap comparison of median CV ratios for random and second base pair stacking groupings 17](#_Toc228227073)

[Supplemental Figure S11: Quantitative analysis of DMS reactivity in WC and non-WC nucleotides 18](#_Toc228227074)

[Supplemental Figure S12: Quantitative analysis of DMS reactivity in flanking Watson-Crick and non-Watson-Crick nucleotides under modified conditions 19](#_Toc228227075)

[Supplemental Figure S13: The reactivity distribution for Flank-WC pairs and non-WC under denaturing conditions 20](#_Toc228227076)

[Supplemental Figure S14: Non-WC and WC overlaps with different normalization methods 21](#_Toc228227077)

[Supplemental Figure S15: Quantitative analysis of reactivity of flanking WC pairs to base pair parameters 22](#_Toc228227078)

[Supplemental Figure S16: Sequence context and structural features influence Watson-Crick pair reactivity under modified conditions 23](#_Toc228227079)

[Supplemental Figure S17: Purines’ preference for stacking interactions over hydrogen bonding in purine-rich environments 24](#_Toc228227080)

[Supplemental Figure S18: Structural and sequence determinants of low reactivity in non-canonical pairs under modified conditions 25](#_Toc228227081)

[Supplemental Figure S19: Correlation between solvent accessible surface area (SASA) and mutation fraction 27](#_Toc228227082)

[Supplemental Figure S20: Impact of neighboring sequences on mismatches 28](#_Toc228227083)

[Supplemental Figure S21: Weaker correlations between non-canonical pairs and DMS reactivity 29](#_Toc228227084)

[Supplemental Figure S22: DMS reactivity correlates with RNA 3D structural features of non-canonical pairs under modified conditions 30](#_Toc228227085)

[Supplemental Figure S23: Correlation plots for distance and reactivity for A-G pairs 31](#_Toc228227086)

[Supplemental Figure S24: Correlation plots for distance and reactivity for C-A pairs 32](#_Toc228227087)

[Supplemental Figure S25: Correlation between cytosine reactivity and atomic distance for C-C mismatches. 33](#_Toc228227088)

[Supplemental Figure S26: Correlation plots for distance and reactivity for C-C pairs 34](#_Toc228227089)

[Supplemental Figure S27: Correlation between interatomic distance and mutation fraction for 1×1 and 2×2 pairs, including newly resolved structures 35](#_Toc228227090)

[Supplemental Figure S28: DMS-informed distance constraints improve Rosetta modeling of RNA motifs with A-G pairs 36](#_Toc228227091)

[Supplemental Figure S29: Validation of optimized DMS-derived distance constraints on independent engineered RNA motifs with A-G pairs 38](#_Toc228227092)

[Supplementary Figure S30: Library design incorporating known two-way junctions with 3D structures and engineered symmetric junctions without 3D structures 39](#_Toc228227093)

[Supplementary Figure S31: Reactive flanking base pairs for C 40](#_Toc228227094)

[Supplemental Table S3: Outliers of the coefficient of variation (CV). 41](#_Toc228227095)

[Supplementary Table S5: DREEM clustering on preliminary constructs 45](#_Toc228227096)

[Supplemental Table S6: Residues under 2 Å for solvent accessible surface area. 46](#_Toc228227097)

## Supplemental Methods: DREEM clustering of example hairpin constructs.

To assess whether structural heterogeneity could account for this variation, we applied the DREEM ensemble deconvolution algorithm (35) to a representative set of constructs analogous to those in our library. While clustering identified two populations, the resulting secondary structures were identical, with minor local differences such as flanking base pair shifts. Only one construct showed significant secondary structural rearrangements, with an alternative structure representing only a minor population (22%). These results suggest that large-scale structural heterogeneity is unlikely to be the main reason for the observed variability (**Supplemental Table S5**).

**
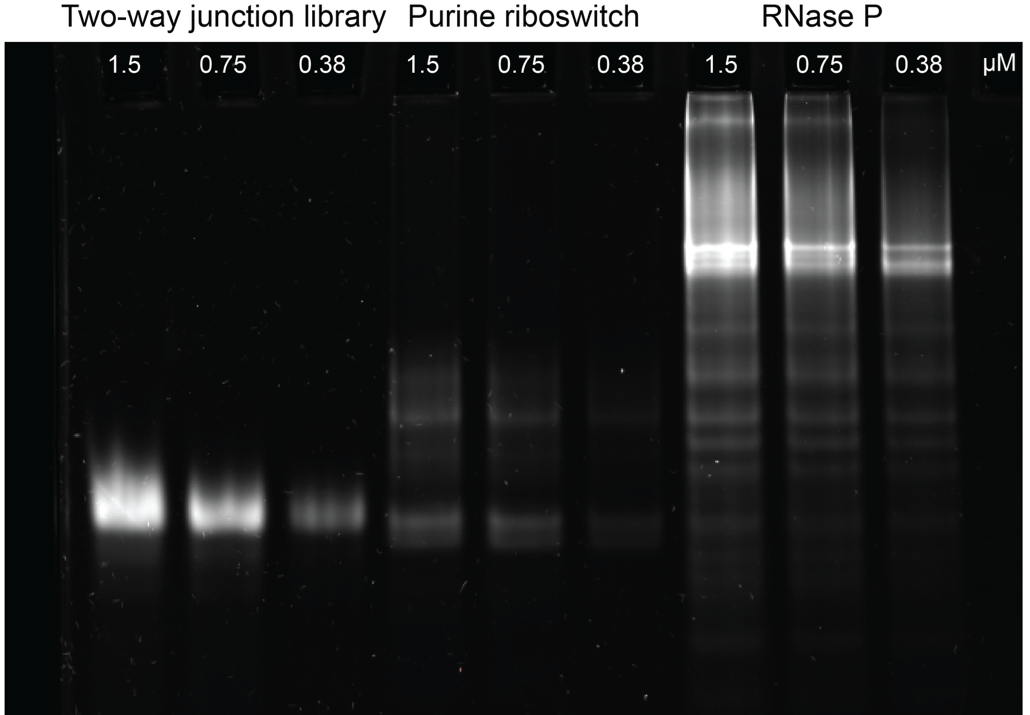
**

## Supplemental Figure S1: Native gel of two-way junction library demonstrates no higher-order interactions

A native gel of the two-way junction library (average length: 145) used in this study, compared to the purine riboswitch (length: 148) and RNase P (length: 347) at three concentrations. The library is slightly larger than the purine riboswitch, which is consistent with its long hairpin compared to the compact purine riboswitch. There are no bands that are consistent with higher-order products in the two-way junction library used in this study.


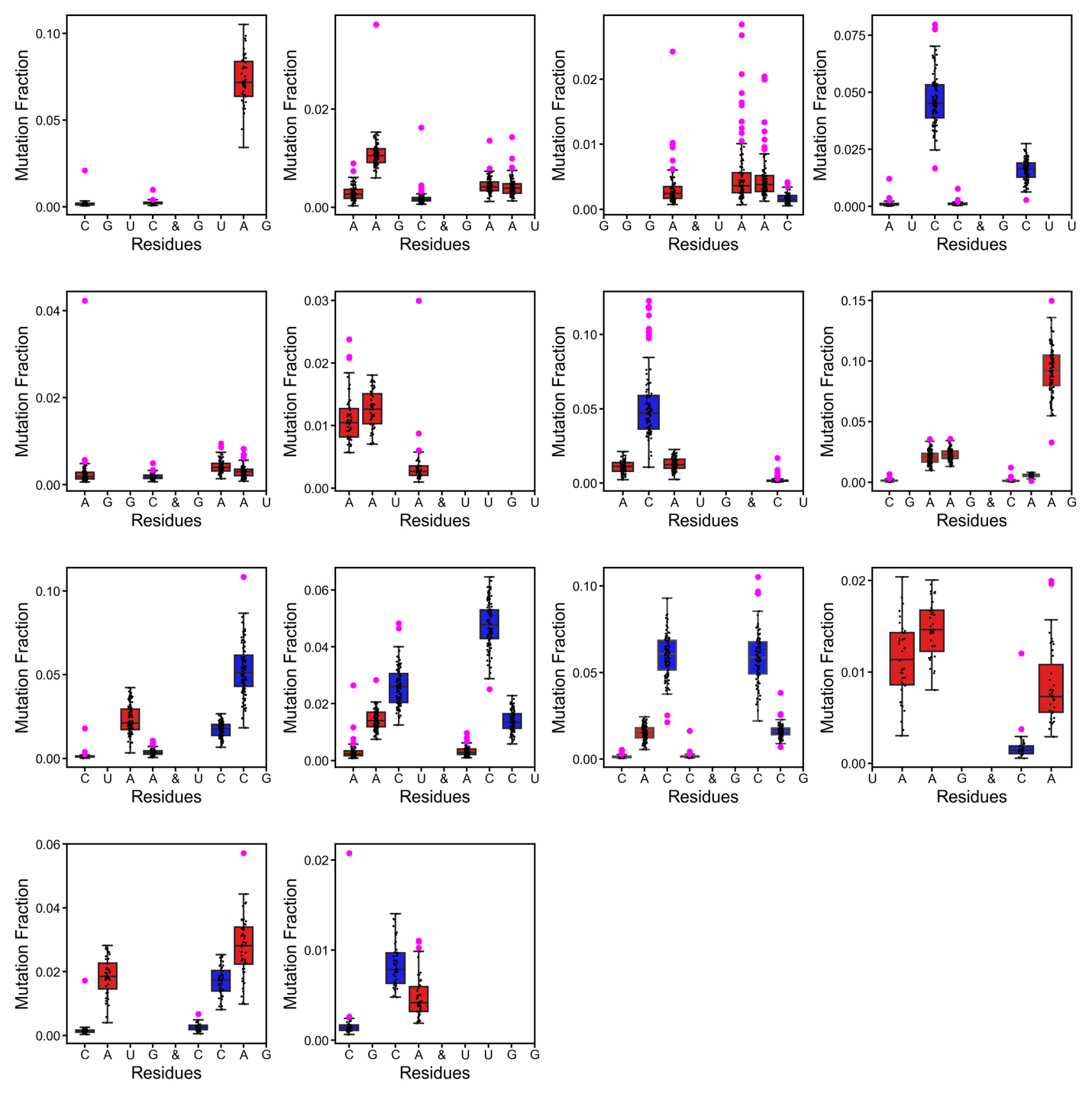


## Supplemental Figure S2: DMS reactivity patterns for motifs highlighting outliers

Box plots depict the reactivity ranges for residues of the few motifs that have outliers, which are defined as points with. The outlier reactivity values are highlighted in magenta. These points were removed for further analysis


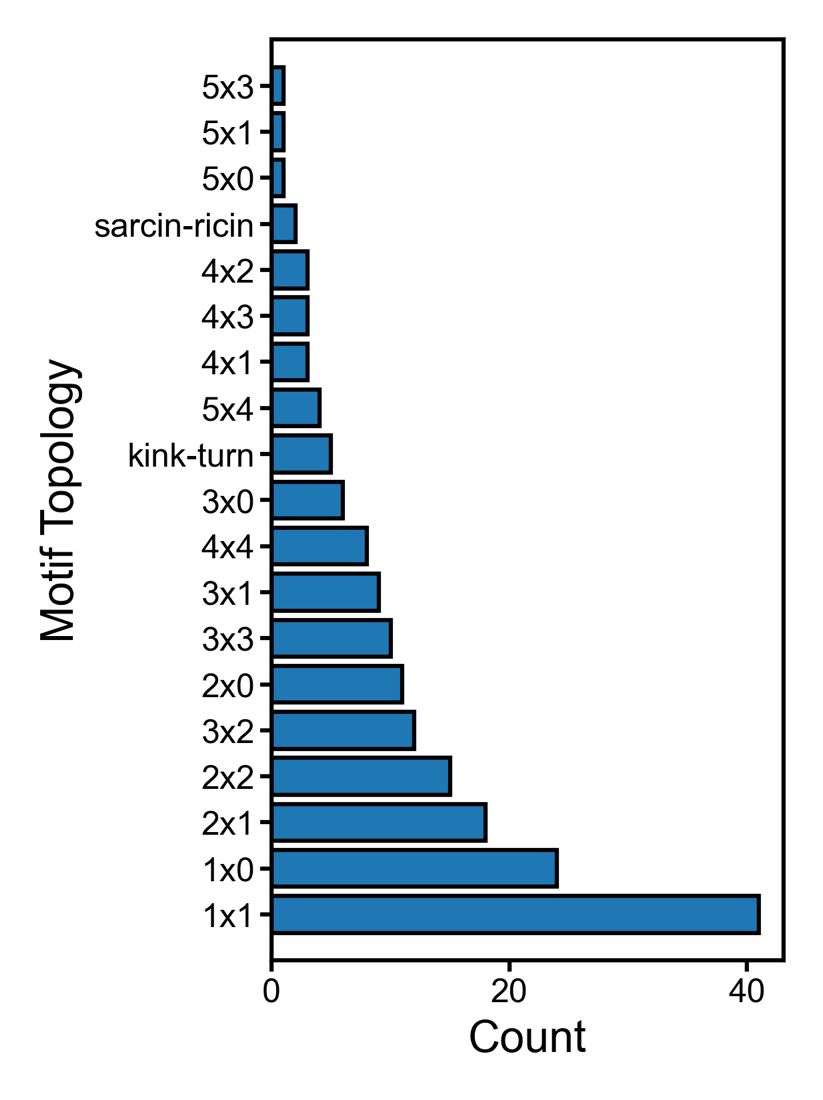


## Supplemental Figure S3: Distribution of two-way junctions

The horizontal bar plot shows the counts of different two-way junctions in the library. The motifs are sorted by their frequency.


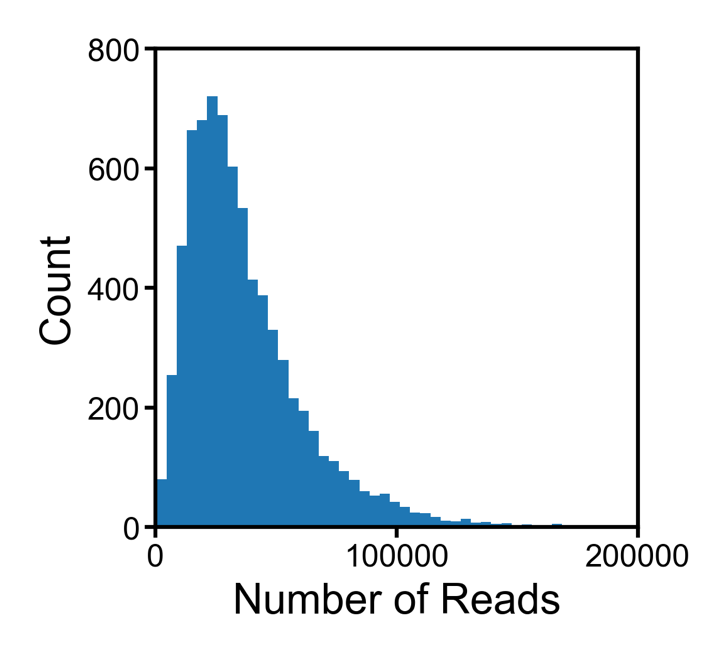


## Supplemental Figure S4: Number of reads per construct

This histogram represents the distribution of the number of reads per construct. Most of the distribution falls between 0 and 100,000 reads. The distribution exhibits a long tail, indicating a small subset of constructs with higher read counts.


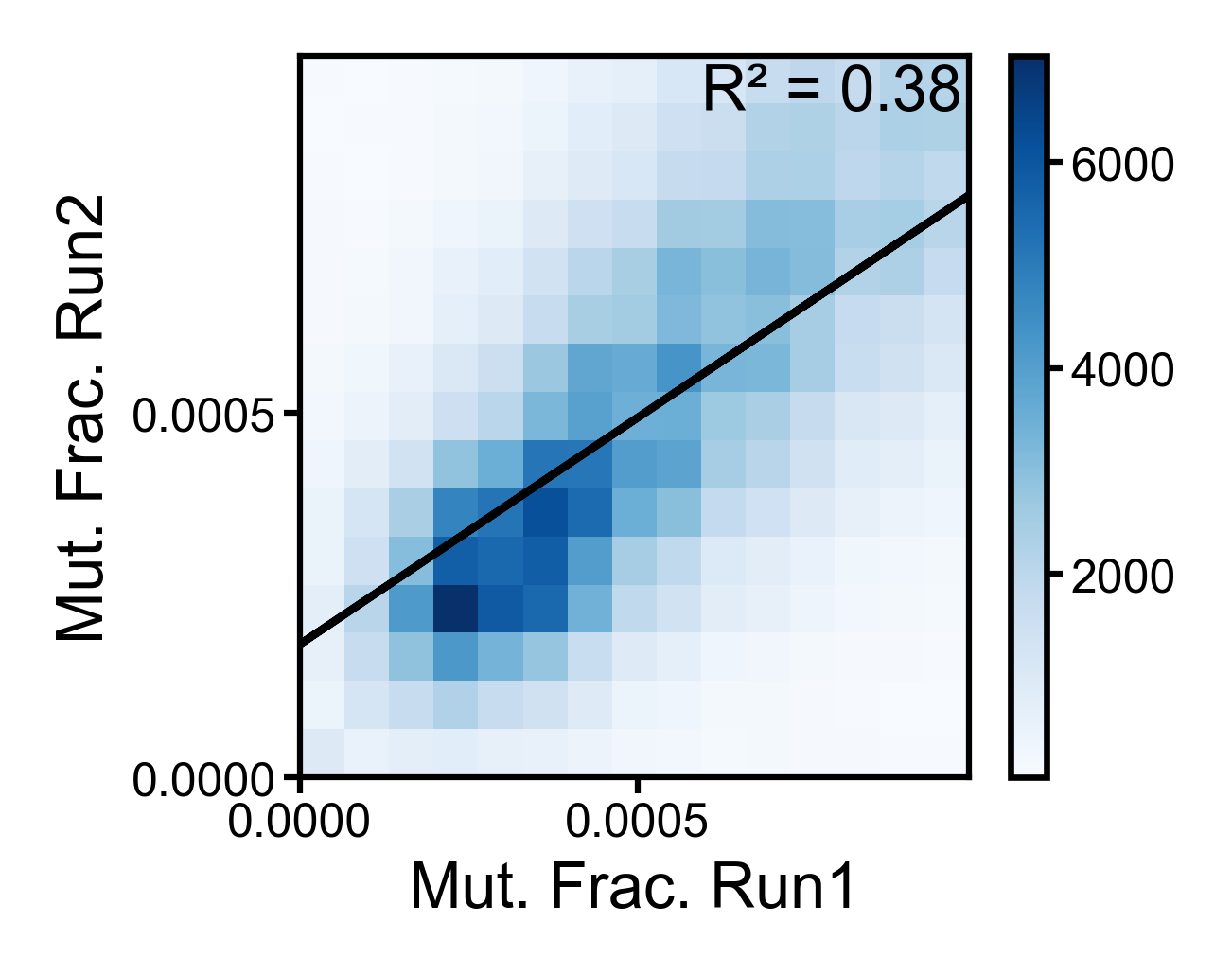


## Supplemental Figure S5: Reproducibility of DMS measurements below 0.001 across independent experiments.

The correlation plot illustrates the R² value between two independent experiments for DMS reactivity values below 0.001. When considering the entire data range, the correlation was strong (R² = 0.99**, Figure 1C**), but it significantly decreased to 0.38 for DMS reactivity values below 0.001**.**


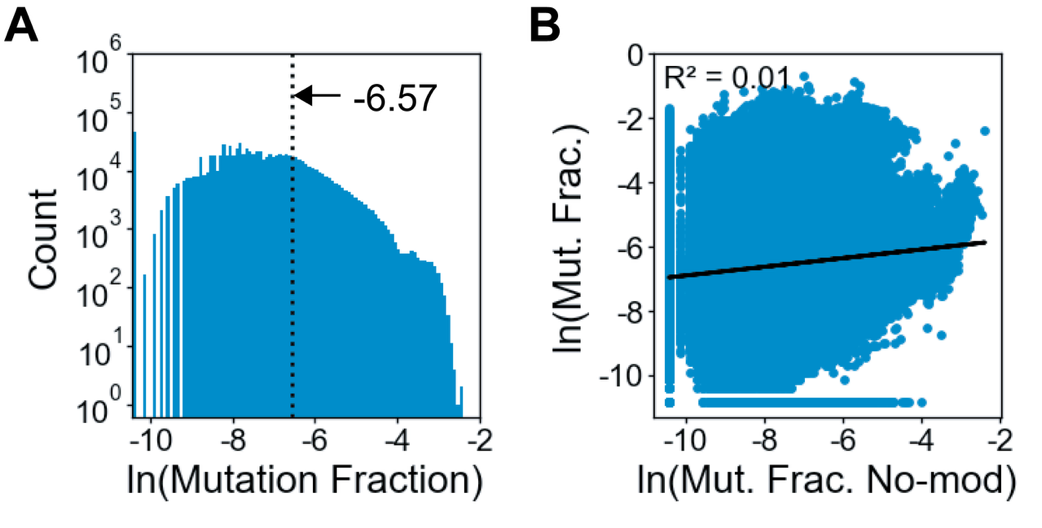


## Supplemental Figure S6: Background mutation rates in the no-modification control are low and uncorrelated with DMS signal

A) Distribution of mutation fractions across all constructs in the no-modification control library. The dotted line indicates the average mutation fraction at −6.57 (reactivity = 0.0014). B) Correlation of mutation fractions under the no-modification conditions versus the DMS-modified library.


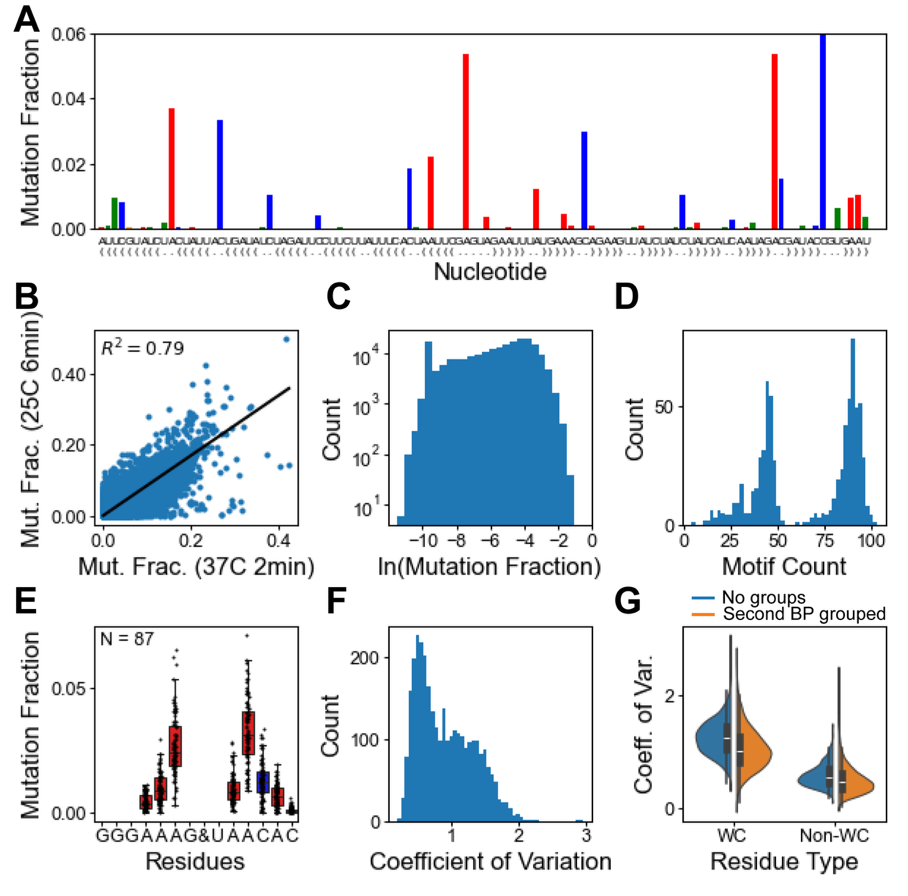


## Supplemental Figure S7: Validation of RNA library for DMS structure-reactivity analysis under modified conditions

Remaking Figure 1 with modified DMS conditions. (A) A representative construct shows the secondary structure and DMS reactivity data. (B) Correlation of reactivity values with modified and original DMS conditions. (C) Distribution of DMS reactivity values shown on a natural logarithmic scale, spanning four orders of magnitude. (E) Frequency distribution of motif occurrences within the library. (F) Example of reactivity consistency: The motif "GGGAAAG&UAACAC" with secondary structure “(…..(&)…..)” exhibits similar DMS reactivity patterns across multiple sequence contexts, demonstrating reproducibility in measurements. (G) Measurement variability analysis: CV for each nucleotide position across all motif instances. (H) Impact of structural context: Comparison of CV distributions when nucleotides are grouped by second flanking pair identity versus ungrouped, showing reduced variability with grouping.


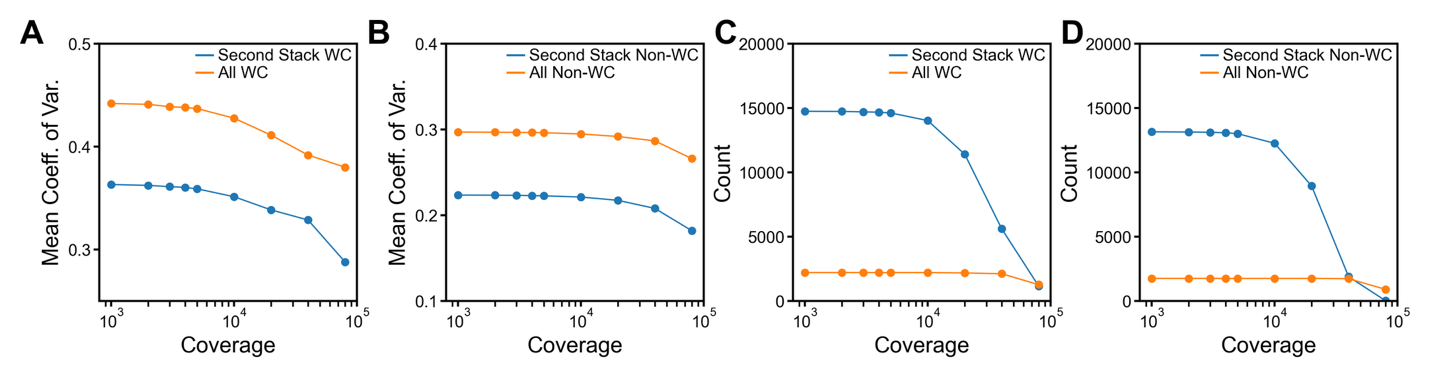


## Supplemental Figure S8: Coverage dependence of the CV for Flank-WC and non-WC residues.

(A) The mean coefficient of variation as a function of the minimum number of reads for flanking WC pairs for all data of a motif averaged compared to those grouped by second base pair flank. (B) Same analysis as in (A), but for non-WC residues. (C–D) The total number of data points included at each coverage threshold for WC and non-WC residues is shown in (A) and (B), respectively. The number of available data points remains high for WC pairs until ∼5000× coverage, after which both categories decline rapidly.


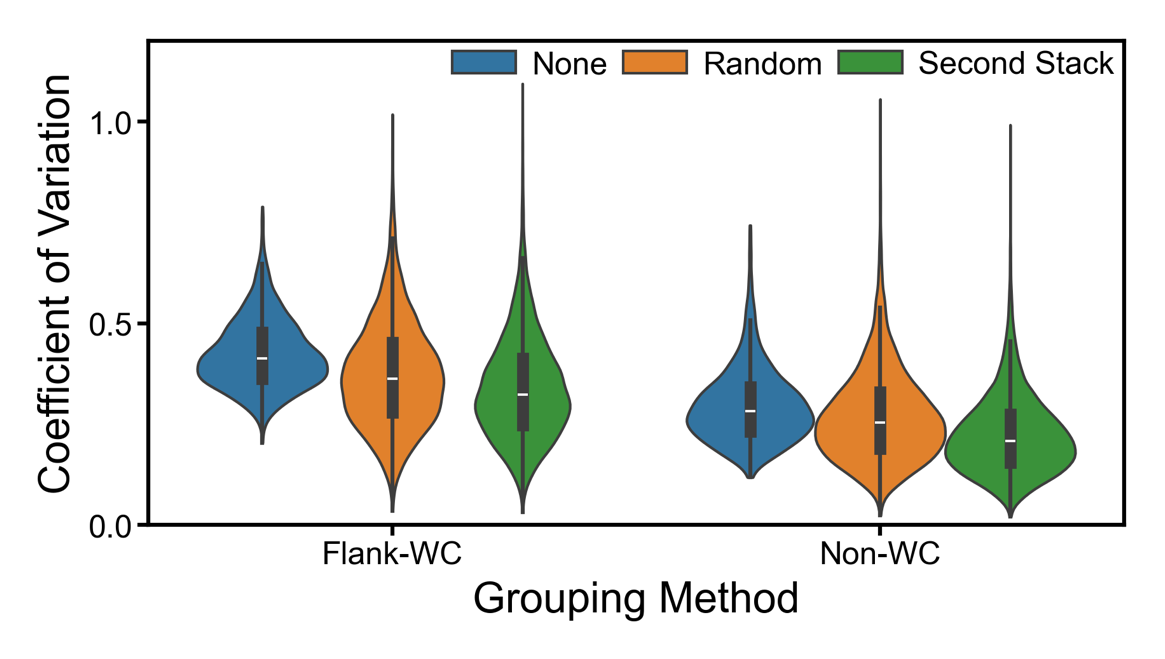


## Supplemental Figure S9: Comparison of variability between second flanking pair grouping and random grouping

Violin plots illustrating the CV across different grouping methods: no grouping (None), random grouping into similarly sized groups (Random), and grouping based on second base pair stacking (Second Stack). These suggest that the smaller grouping is not sufficient to explain the decrease in CV by second stack.


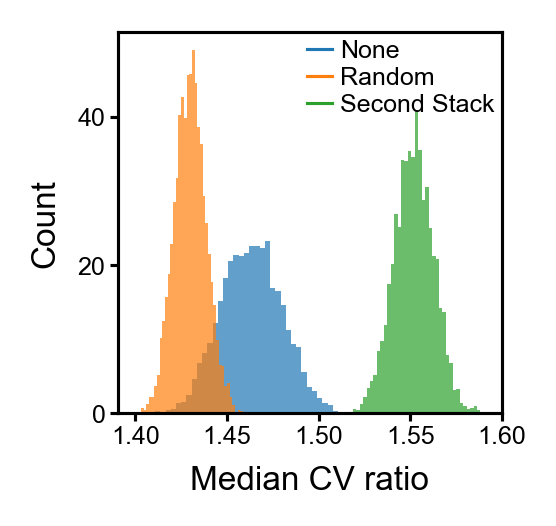


## Supplemental Figure S10: Bootstrap comparison of median CV ratios for random and second base pair stacking groupings

Bootstrap distributions of the median coefficient of variation (CV) ratios for the ungrouped (None), Random, and Second Stack grouping methods. Each distribution represents 10,000 resampled estimates of the median CV ratio. The ungrouped (blue) and Random (orange) distributions overlap closely, indicating comparable variability across resamples. In contrast, the Second Stack grouping (green) shows a clearly shifted distribution toward higher CV ratios, demonstrating that grouping by second base pair stacking results in systematically greater variability compared to random or ungrouped data.


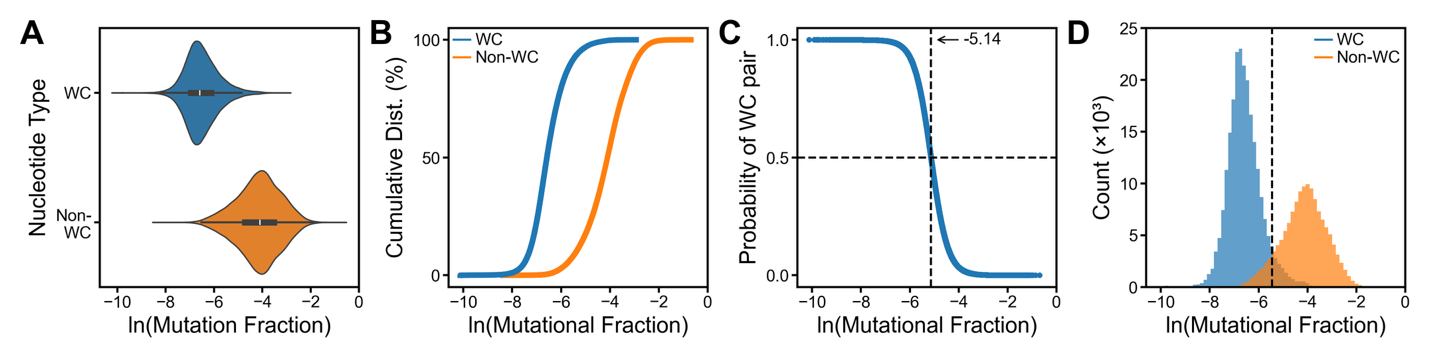


## Supplemental Figure S11: Quantitative analysis of DMS reactivity in WC and non-WC nucleotides

Remaking Figure 2, including all WC pairs, not just flanking pairs, the results are similar to just including flanking WC pairs (A) Reactivity distribution of Non-WC and WC nucleotides. (B) Cumulative reactivity distributions comparing WC paired versus non-WC nucleotides as a function of the natural log of the mutational histogram. (C) The logistic regression analysis establishes the probability of WC pairing based on DMS reactivity. The horizontal dashed line marks the 50% probability threshold, corresponding to a natural log mutation fraction of -5.19 (mutation fraction = 0.0056). (D) Distribution of nucleotides relative to the 50% probability threshold.


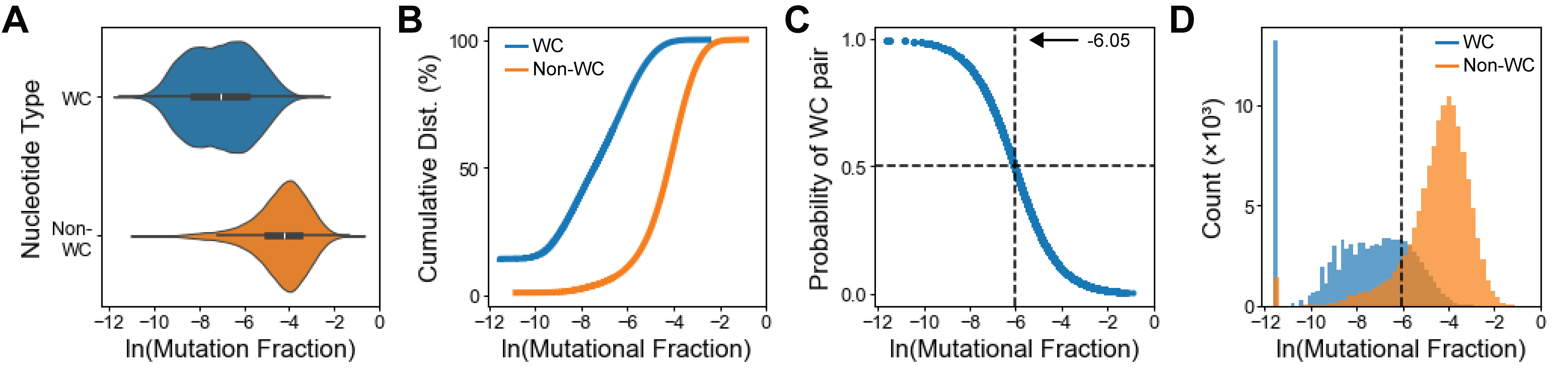


## Supplemental Figure S12: Quantitative analysis of DMS reactivity in flanking Watson-Crick and non-Watson-Crick nucleotides under modified conditions

Remaking Figure 2 with modified DMS conditions. (A) Reactivity distributions reveal a significant overlap between nucleotides in flanking WC pairs (blue) and non-WC positions (orange). (B) Cumulative reactivity distributions comparing WC paired versus non-WC nucleotides as a function of the natural log of the mutational histogram. (C) Logistic regression analysis establishing the probability of WC pairing based on DMS reactivity. The horizontal dashed line marks the 50% probability threshold, corresponding to a natural log mutation fraction of -6.05 (mutation fraction = 0.0024). (D) Distribution of nucleotides relative to the 50% probability threshold. The distributions are left-skewed due to the presence of zero-mutation residues, causing the threshold line to deviate from the intersection point.


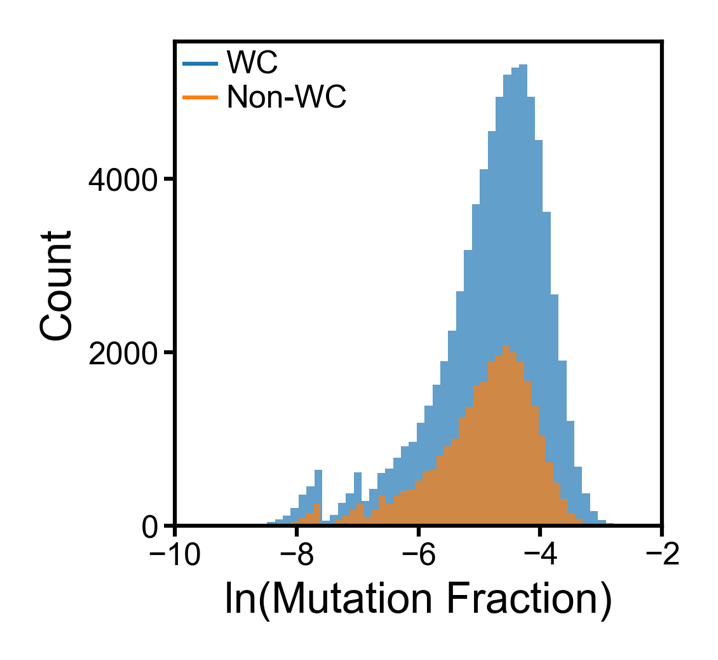


## Supplemental Figure S13: The reactivity distribution for Flank-WC pairs and non-WC under denaturing conditions

Distributions of mutation fraction for Flank-WC and non–WC base pairs collected at 90 °C and in 7 M urea. Under these conditions, the previously observed bimodal distribution of mutation fractions, separating Flank-WC and non-WC nucleotides, collapsed into a single peak, indicating the RNA has largely denatured.


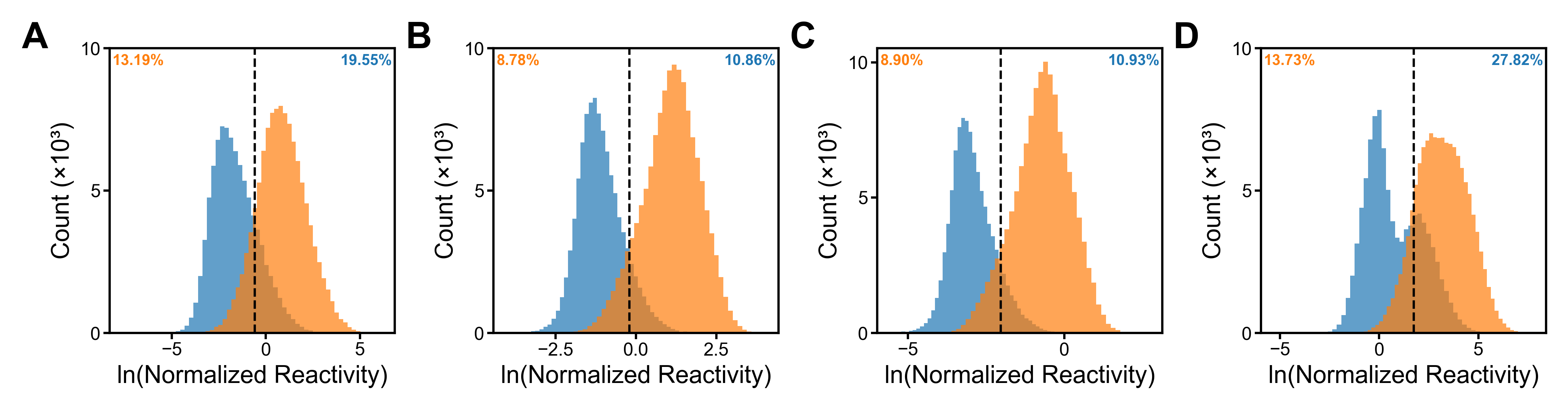


## Supplemental Figure S14: Non-WC and WC overlaps with different normalization methods

Effect of different normalization methods on the distribution of DMS reactivity for Flank-WC and non-WC base pairs. (A) Normalization by reactivities measured under denaturing conditions. (B) Normalization by taking the average reactivity per construct. (C) Normalization by the reactivity of the two adenines in the reference hairpin (GCGAGUAGC). (D) Normalization by reactivities from the no-modification dataset (**see Methods**).


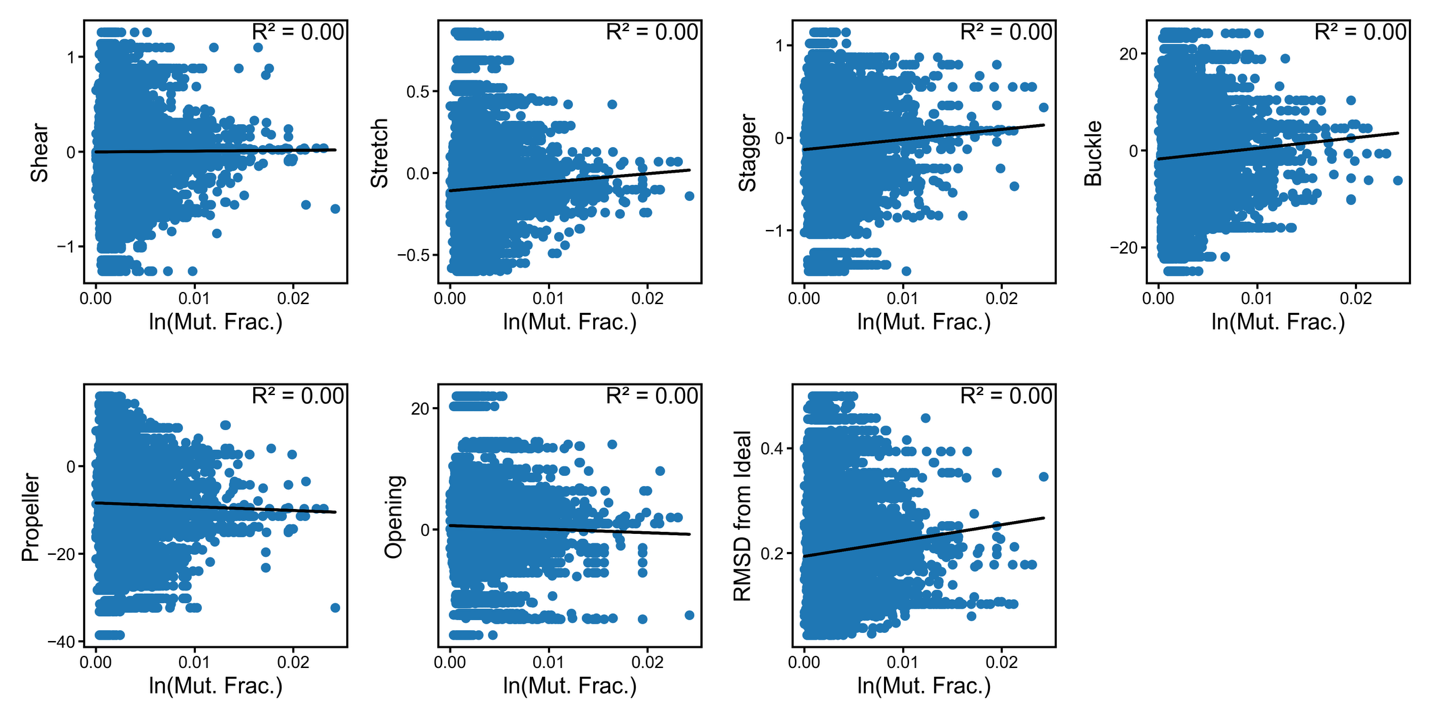


## Supplemental Figure S15: Quantitative analysis of reactivity of flanking WC pairs to base pair parameters

Scatter plots illustrating the correlation between the natural logarithm of DMS reactivity and the six base pair parameters: shear, stretch, stagger, buckle, propeller, and opening. R2 values indicate no correlation between the reactivity and the parameters.


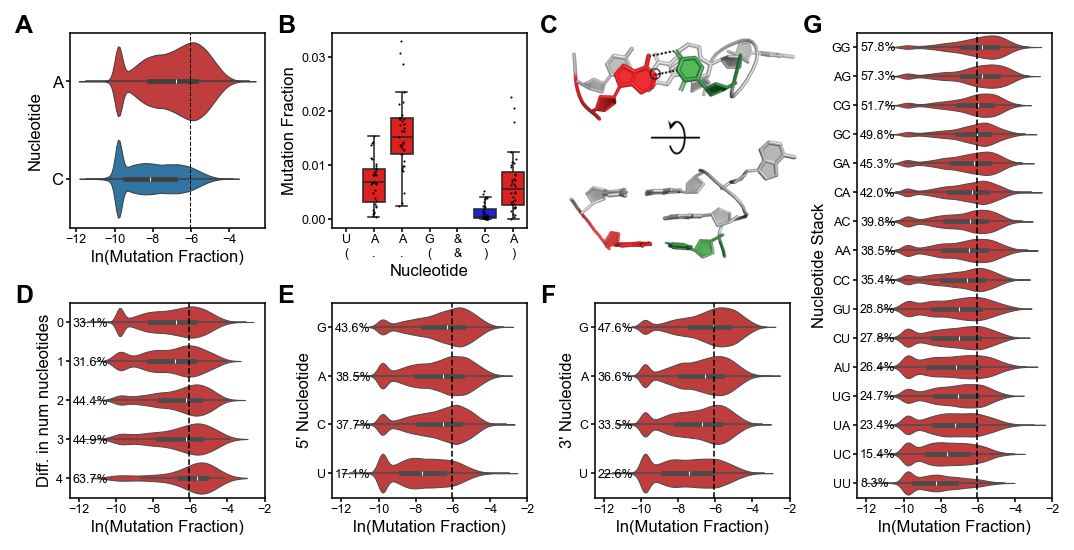


## Supplemental Figure S16: Sequence context and structural features influence Watson-Crick pair reactivity under modified conditions

Remaking Figure 3 with modified DMS conditions. (A) DMS reactivity distributions comparing adenines in A-U pairs versus cytosines in C-G pairs. The vertical dotted line (natural log of reactivity = -6.05) is the 50% likelihood of being a WC base pair. A-U pairs are frequently more reactive than C-G pairs. (B-C) Example snapshot of a WC pair exhibiting high reactivity. This static structure represents only one conformation and does not capture the full range of possible configurations or dynamic behavior. (D) Distributions of the natural log of reactivity for As in A-U flanking pairs as a function of the asymmetry of the non-WC paired residues. (E) The distribution of the natural log of reactivity as a function of the 5′ residue or the residue that appears right before the A in the A-U pair. (F) The distribution of the natural log of reactivity as a function of the 3′ residue or the residue that appears right after the A in the A-U pair. (G) The combined influence of flanking sequence context. Two-nucleotide patterns (e.g., "GG" = 5′-GAG-3′) reveal strong neighboring effects. High distribution around -10 is due to zero-mutation residues.


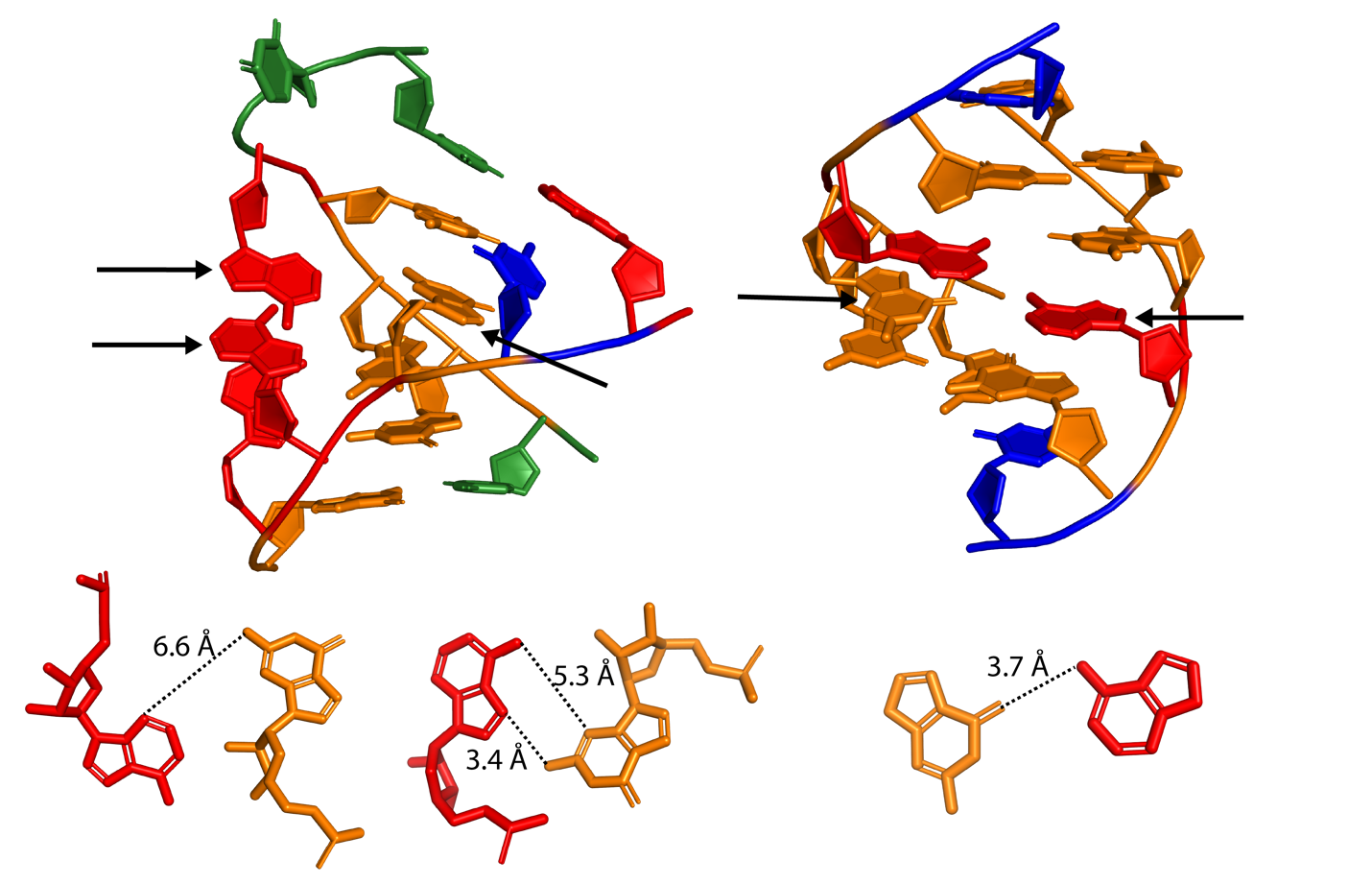


## Supplemental Figure S17: Purines’ preference for stacking interactions over hydrogen bonding in purine-rich environments

This figure depicts the structural preferences of purines in purine-rich environments, where stacking interactions are favored over hydrogen bonding. The top panels show molecular arrangements highlighting purine stacking interactions with arrows showing the regions of interest. The bottom panels present the measured distances between purine bases, demonstrating that these distances exceed the typical hydrogen bond length (< 3.3 Å), emphasizing the dominance of stacking interactions in these configurations. These are two examples from the library, representing instances of purine stacking behavior.


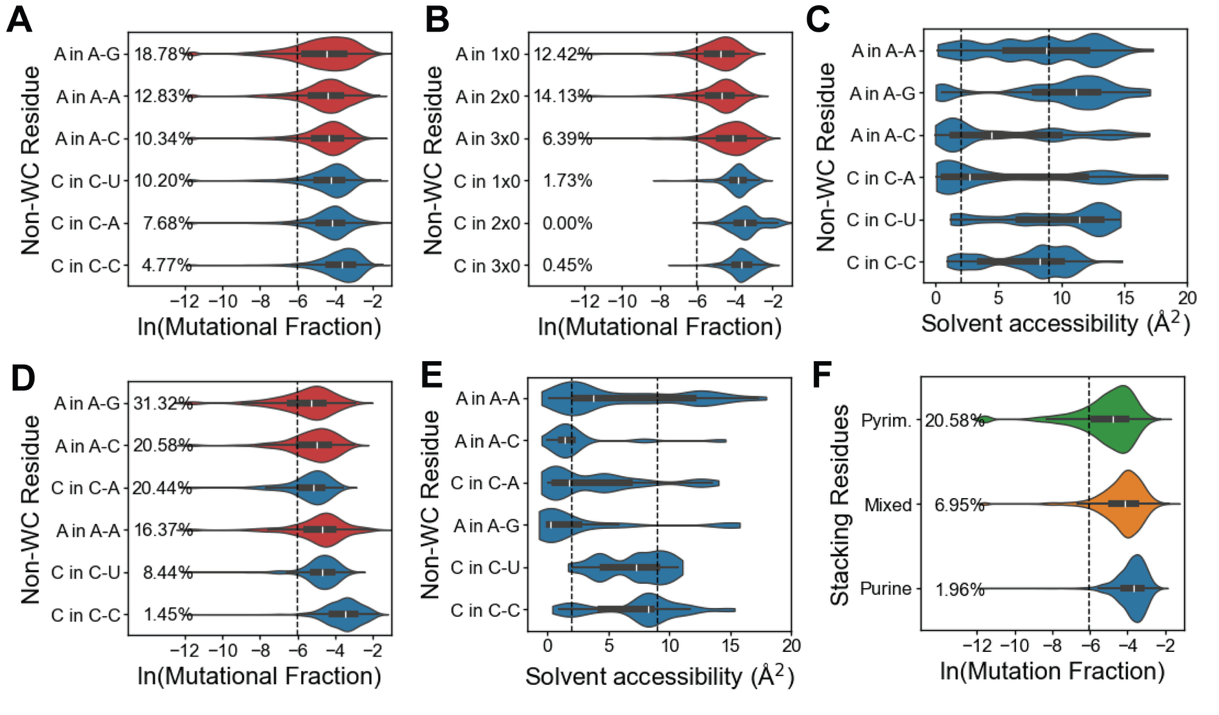


## Supplemental Figure S18: Structural and sequence determinants of low reactivity in non-canonical pairs under modified conditions

Remaking Figure 4 with modified DMS conditions. (A) DMS reactivity distributions for non-canonical pairs with known structures. A vertical dotted line indicates the 50% WC probability threshold (-6.05). Nucleotides left of this line exhibit WC-like protection. (B) Reactivity distribution of unpaired nucleotides in bulges, providing baseline comparison for non-canonical pairs. (C) Solvent accessibility of DMS modification sites across non-canonical pairs. Vertical lines indicate average accessibility for WC pairs (2 Å^2^ left) and unpaired nucleotides (9 Å^2^, right), demonstrating a correlation between accessibility and reactivity. (D) DMS reactivity distributions for non-canonical pairs with known structures in 1x1 mismatches. A vertical dotted line indicates the 50% WC probability threshold (-6.05). Nucleotides left of this line exhibit WC-like protection. (E) Solvent accessibility analysis focused on 1×1 mismatches, revealing distinct patterns of nucleotide protection in symmetric contexts. (F) The impact of neighboring sequences on C-U mismatch reactivity shows how local context modulates DMS accessibility in non-canonical pairs.


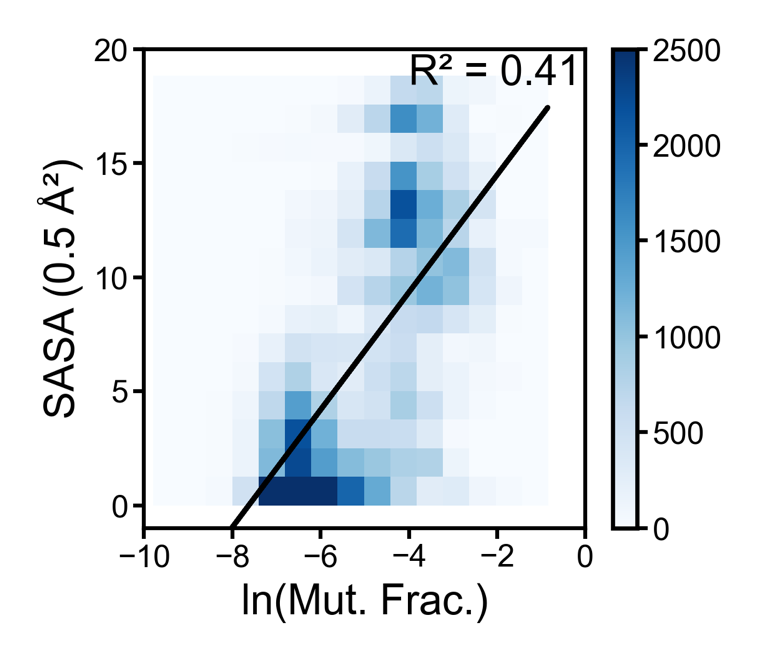


## Supplemental Figure S19: Correlation between solvent accessible surface area (SASA) and mutation fraction

This heat map shows the relationship between the solvent accessible surface area (measured at 0.5 Å probe radius) and the natural logarithm of the mutation fraction. The linear regression line demonstrates a moderate positive correlation, with an R² value of 0.41.


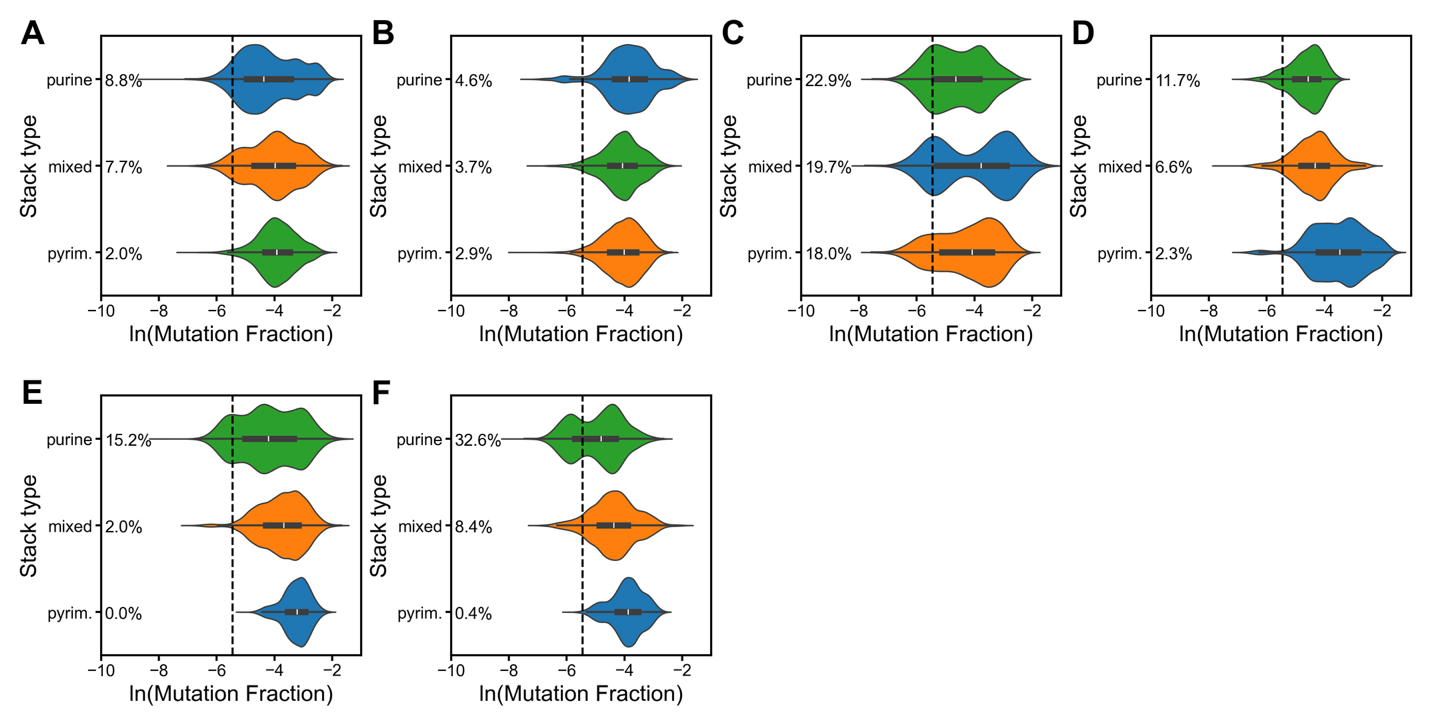


## Supplemental Figure S20: Impact of neighboring sequences on mismatches

Violin plots showing the effect of neighboring nucleotide sequence context on mismatch mutation fractions. Each panel (A–F) represents the distribution of ln(Mutation Fraction) for specific neighboring base pair contexts: (A) A–A, (B) A–C, (C) A–G, (D) C–A, (E) C–C, and (F) C–U. Data are grouped by stack type: purine, mixed, and pyrimidine. The horizontal dashed line denotes the ln(Mutation Fraction) threshold of –5.45, corresponding to a 50% probability boundary of being a WC pair. Percentages next to each category indicate the proportion of data points with ln(Mutation Fraction) < –5.45.


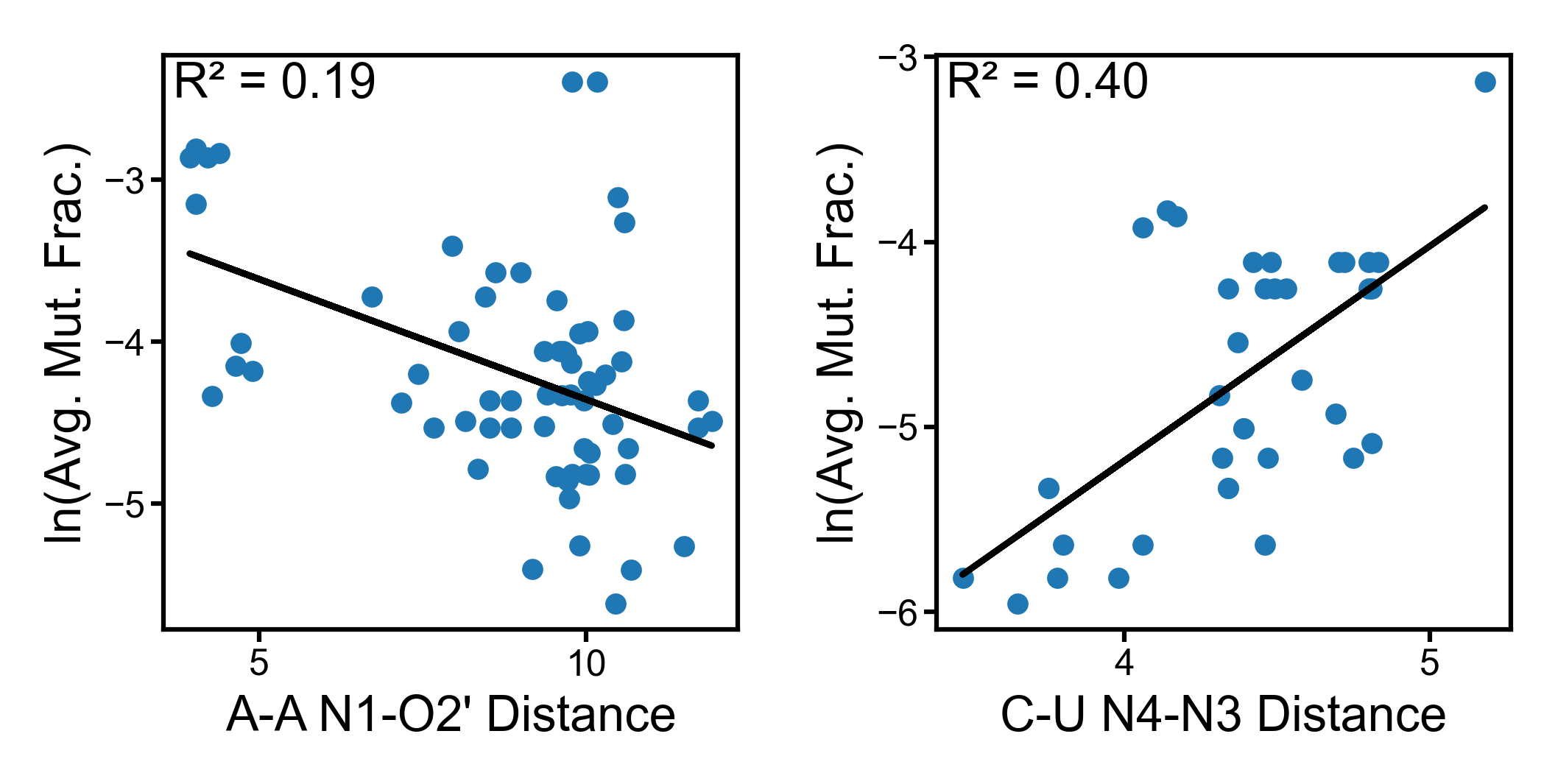


## Supplemental Figure S21: Weaker correlations between non-canonical pairs and DMS reactivity

Scatter plots showing the relationship between DMS reactivity and atomic distances in non-canonical base pairs. Each plot represents a specific atom pair: (left) A–A N1–O2′ and (right) C–U N4–N3. The linear regression lines show modest positive or negative correlations, with R² values of 0.19 and 0.40, respectively, indicating weaker associations compared to the other non-canonical pairs. Reactivity is expressed as the natural logarithm of the average mutational fraction for each mismatch.

##
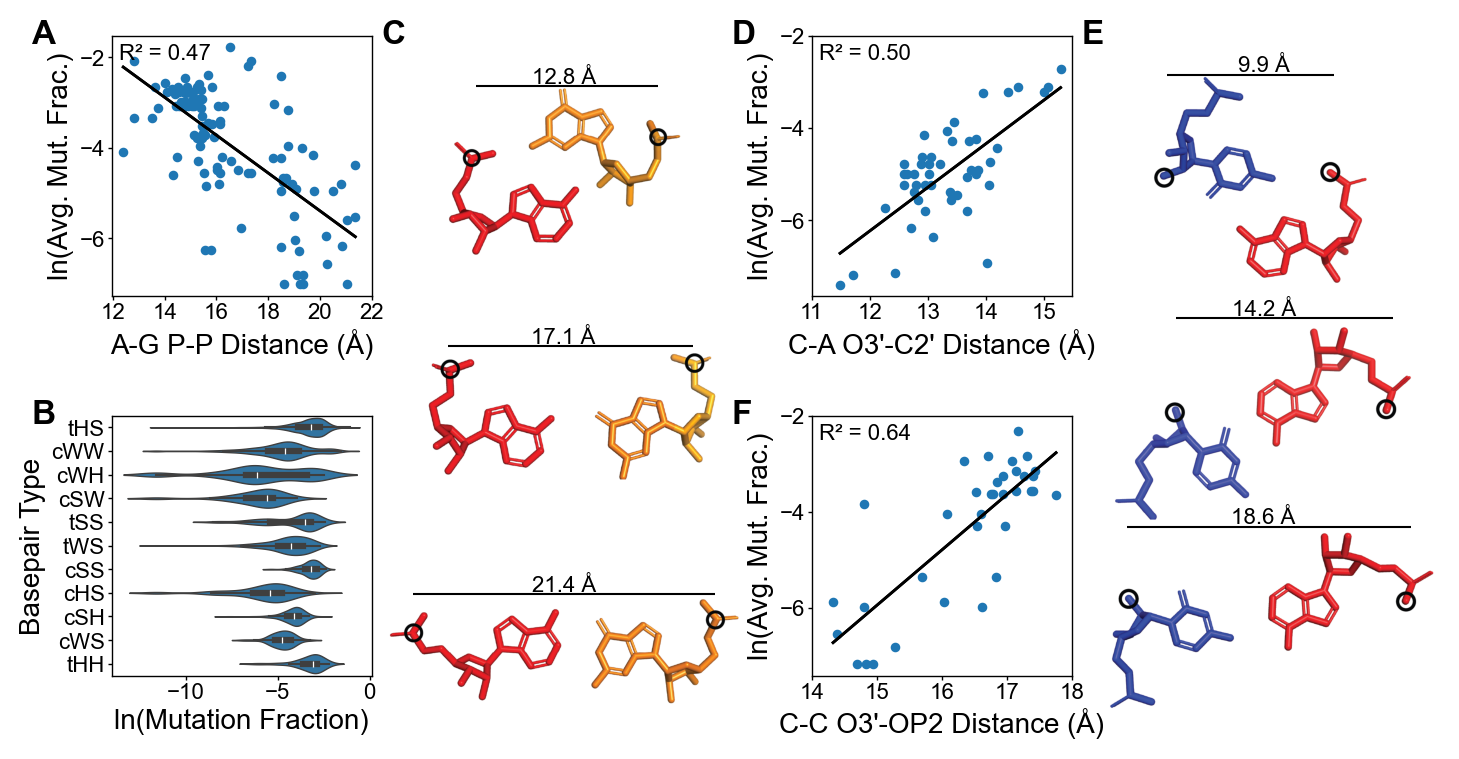


## Supplemental Figure S22: DMS reactivity correlates with RNA 3D structural features of non-canonical pairs under modified conditions

Remaking Figure 5 with modified DMS conditions. (A) The natural log of adenine reactivity in A-G pairs correlates with phosphate-phosphate distance (R² = 0.47). (B) Representative A-G pairs show short, medium, and long P-P distances. (C) Distribution of adenine reactivity in A-G pairs by base pair conformation type, showing distinct patterns. (D) Cytosine reactivity in C-A pairs correlates with O3′-C2′ distance (R² = 0.50). (E) Representative C-A pairs show short, medium, and long O2′-OP2 distances. (F) The cytosine reactivity in C-C pairs correlates with O3′-OP2 distances (R² = 0.64).


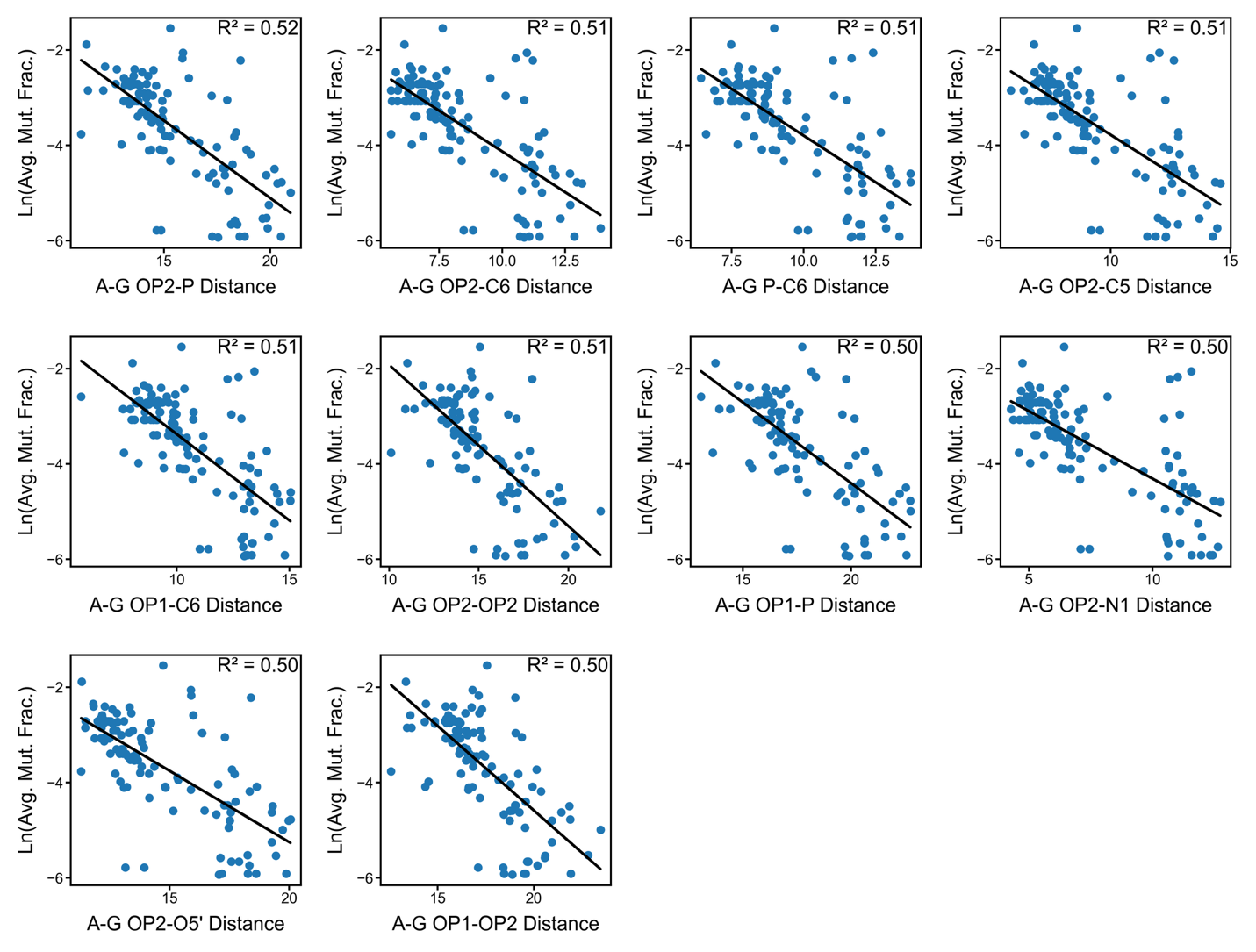


## Supplemental Figure S23: Correlation plots for distance and reactivity for A-G pairs

Scatter plots showing the top 10 correlations between atomic distances in A-G pairs and the natural logarithm of DMS reactivity of the A. Each plot represents a specific atomic pair distance (e.g., OP2-P, OP2-C6, OP1-C6), with the corresponding R² values indicating the strength of the correlation.


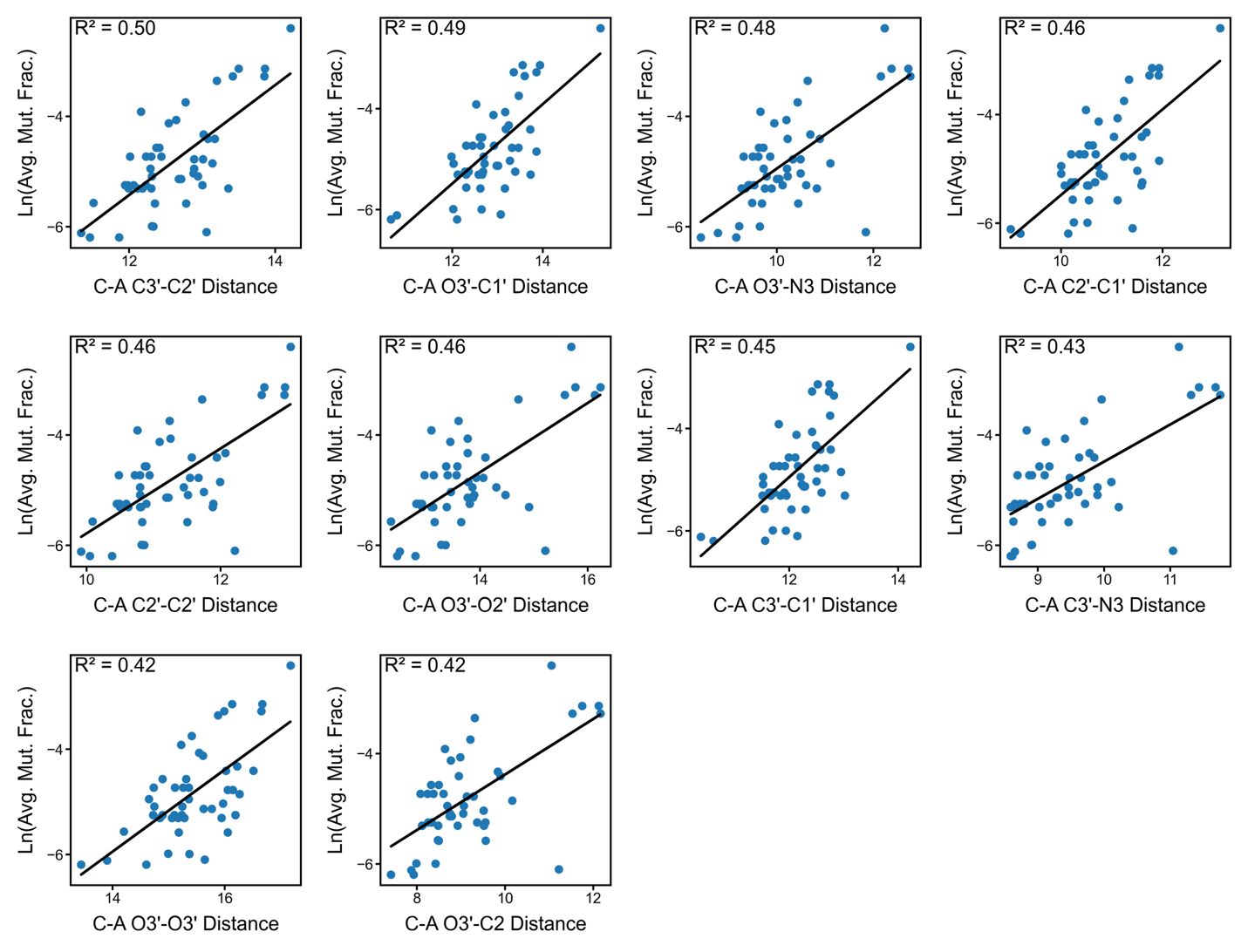


## Supplemental Figure S24: Correlation plots for distance and reactivity for C-A pairs

Scatter plots showing the top 10 correlations between atomic distances of C in C-A pairs and the natural logarithm of DMS reactivity. Each plot represents a specific atomic pair distance (e.g., C3′-C2′, O3′-C1′, O3′-N3), with the corresponding R² values indicating the strength of the correlation.


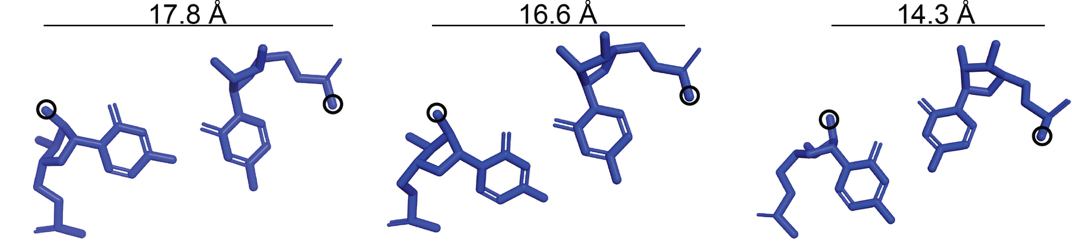


## Supplemental Figure S25: Correlation between cytosine reactivity and atomic distance for C-C mismatches.

Representative structural models of C-C mismatches with varying O3’–OP2’ distances (17.8 Å, 16.6 Å, and 14.3 Å), showing the spatial arrangement of cytosine residues.


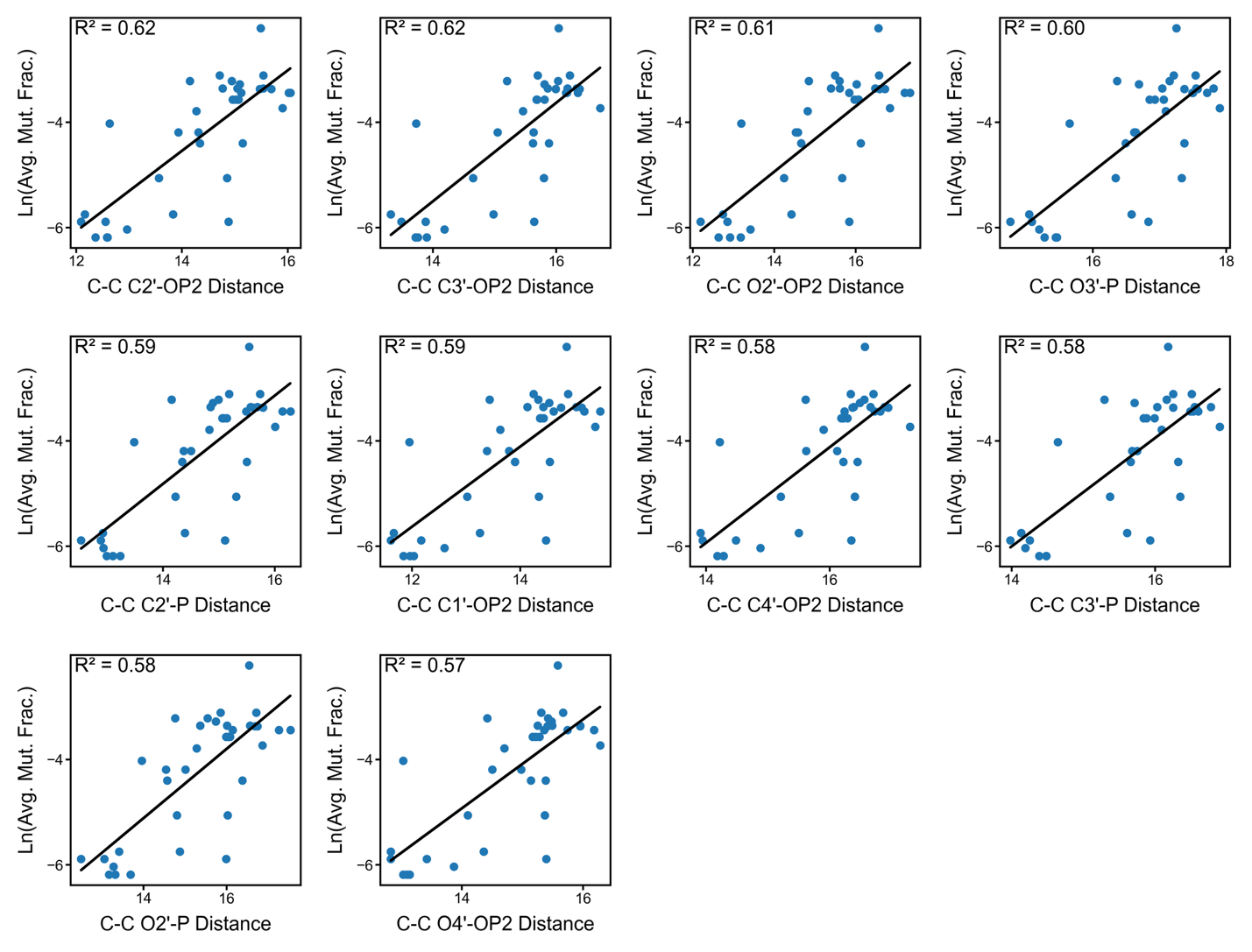


## Supplemental Figure S26: Correlation plots for distance and reactivity for C-C pairs

Scatter plots showing the top 10 correlations between interatomic distances in C-C pairs and the natural logarithm of DMS reactivity. Each plot represents a specific atomic pair distance (e.g., C2′-OP2, C3′-OP2, O2′-OP2), with the corresponding R² values indicating the strength of the correlation.


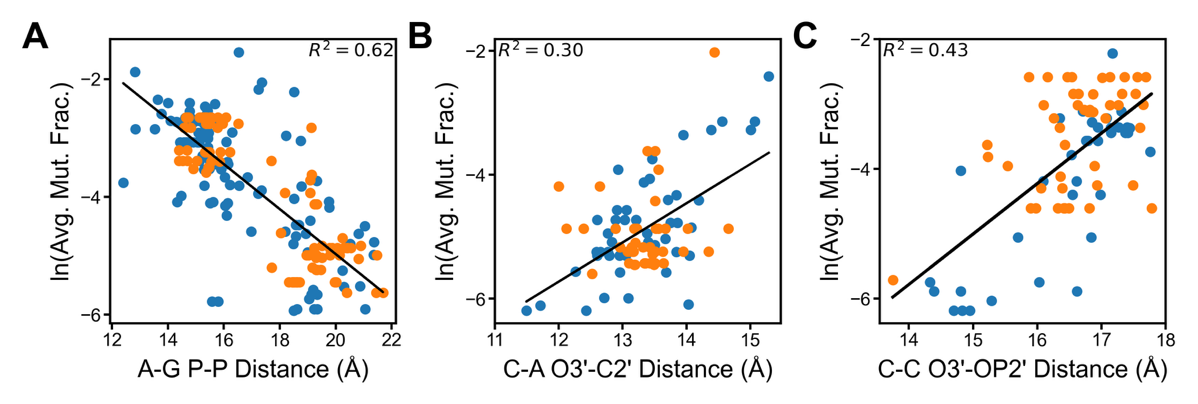


## Supplemental Figure S27: Correlation between interatomic distance and mutation fraction for 1×1 and 2×2 pairs, including newly resolved structures

Scatter plots showing the relationship between interatomic distances and the natural logarithm of the average mutation fraction for 1×1 and 2×2 mismatches. Blue points represent mismatches with previously determined 3D structures, while orange points correspond to mismatches newly resolved in recent crystal or cryo-EM structures. Each plot (A–C) shows the linear regression fit with the corresponding R² value indicating the strength of correlation. The newly added data follow trends consistent with the previously established relationships, reinforcing that these correlations are broadly predictive across expanded structural datasets.


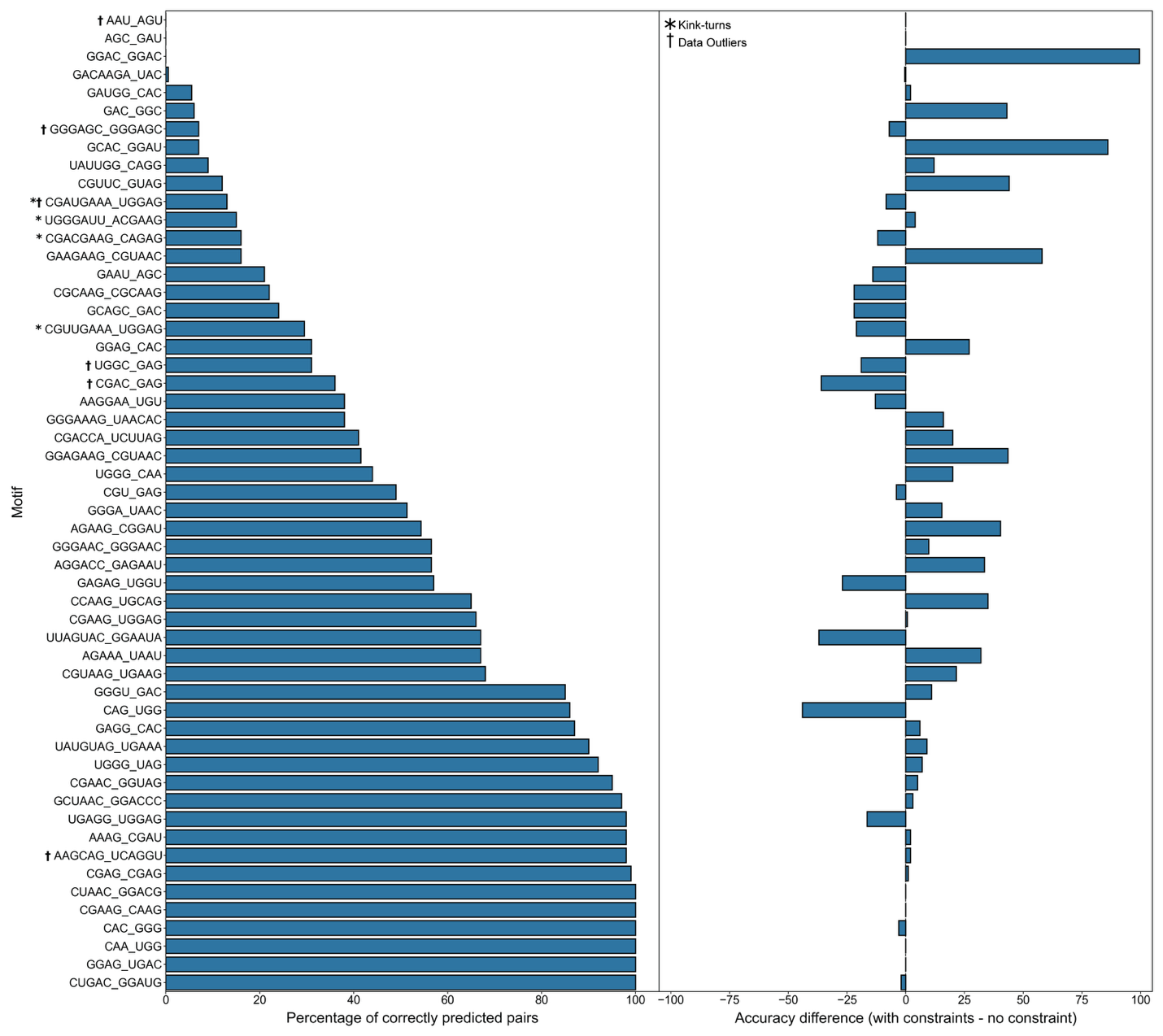


## Supplemental Figure S28: DMS-informed distance constraints improve Rosetta modeling of RNA motifs with A-G pairs

Bar plots showing modeling performance for RNA motifs containing A-G pairs using Rosetta FARFAR. Motifs are sorted by prediction accuracy without constraints (left panel), measured as the percentage of correctly predicted base-pair types. The right panel shows the change in prediction accuracy upon applying distance constraints derived from DMS reactivity. Positive values indicate improved performance with constraints, while negative values indicate reduced accuracy. Motifs marked with (†) denote statistical outliers based on residual analysis, and those marked with (*) indicate kink-turn motifs. A dashed vertical line indicates no change in accuracy.


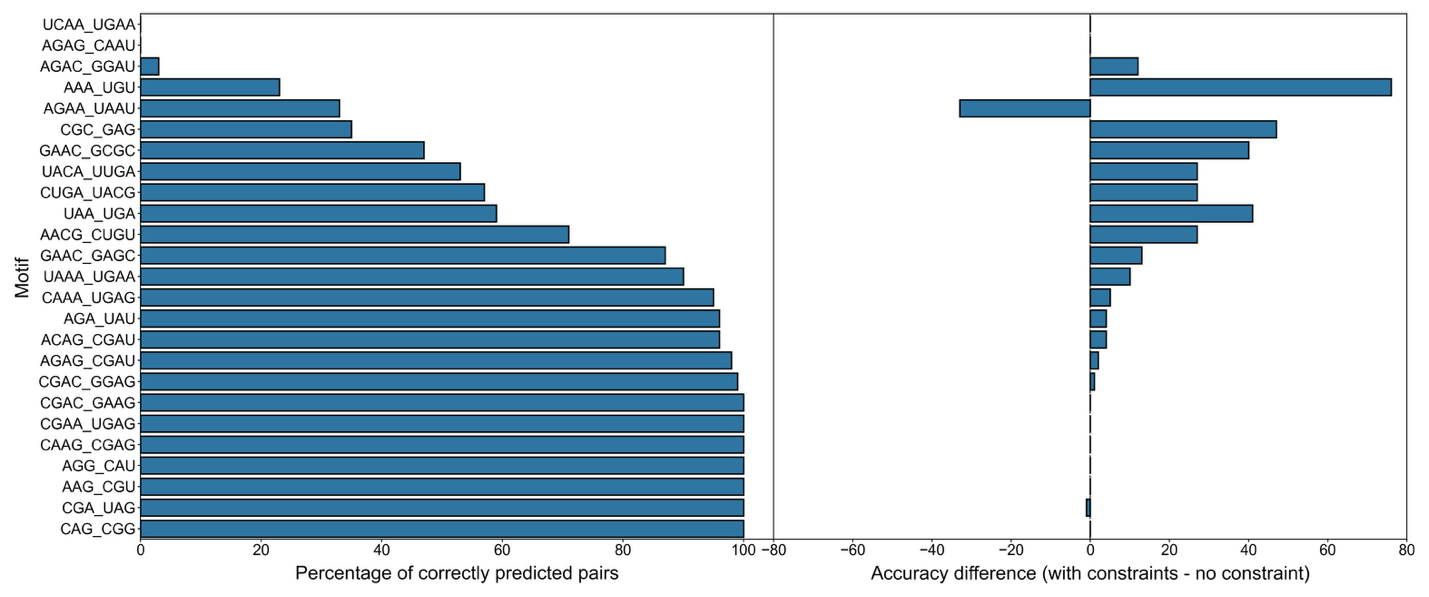


## Supplemental Figure S29: Validation of optimized DMS-derived distance constraints on independent engineered RNA motifs with A-G pairs

Bar plots showing modeling performance for engineered 1×1 and 2×2 RNA motifs containing A-G pairs using Rosetta FARFAR. Motifs are sorted by prediction accuracy without constraints (left panel), measured as the percentage of correctly predicted base-pair types. The right panel shows the change in prediction accuracy after applying distance constraints derived from DMS reactivity, with positive values indicating improved performance and negative values indicating reduced accuracy.


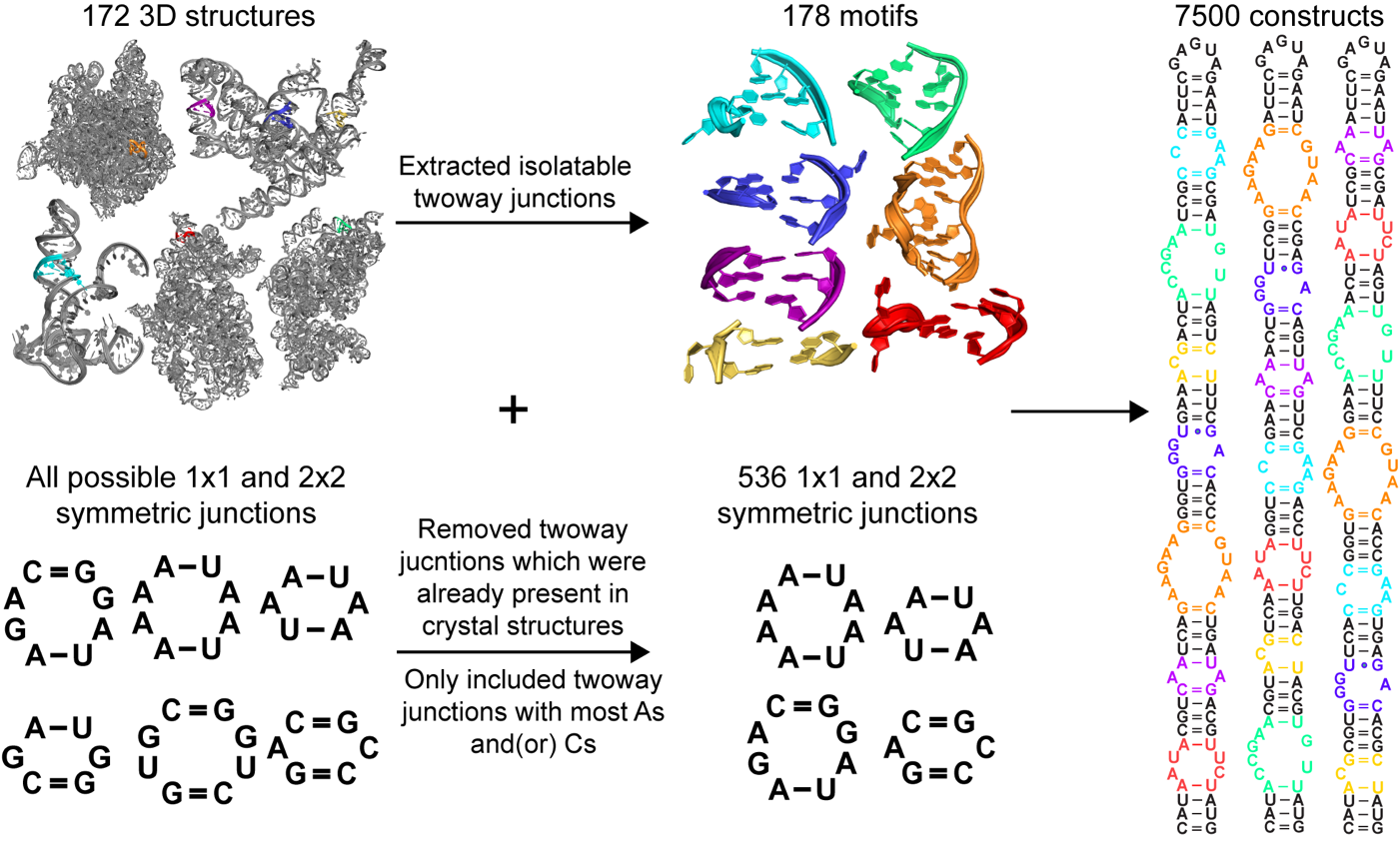


## Supplementary Figure S30: Library design incorporating known two-way junctions with 3D structures and engineered symmetric junctions without 3D structures

The overall library design strategy is shown in **Figure 1A**. This figure specifically illustrates how both known two-way junctions with known 3D structures and engineered 1×1 and 2×2 symmetric two-way junctions without known 3D structures were incorporated into the library.


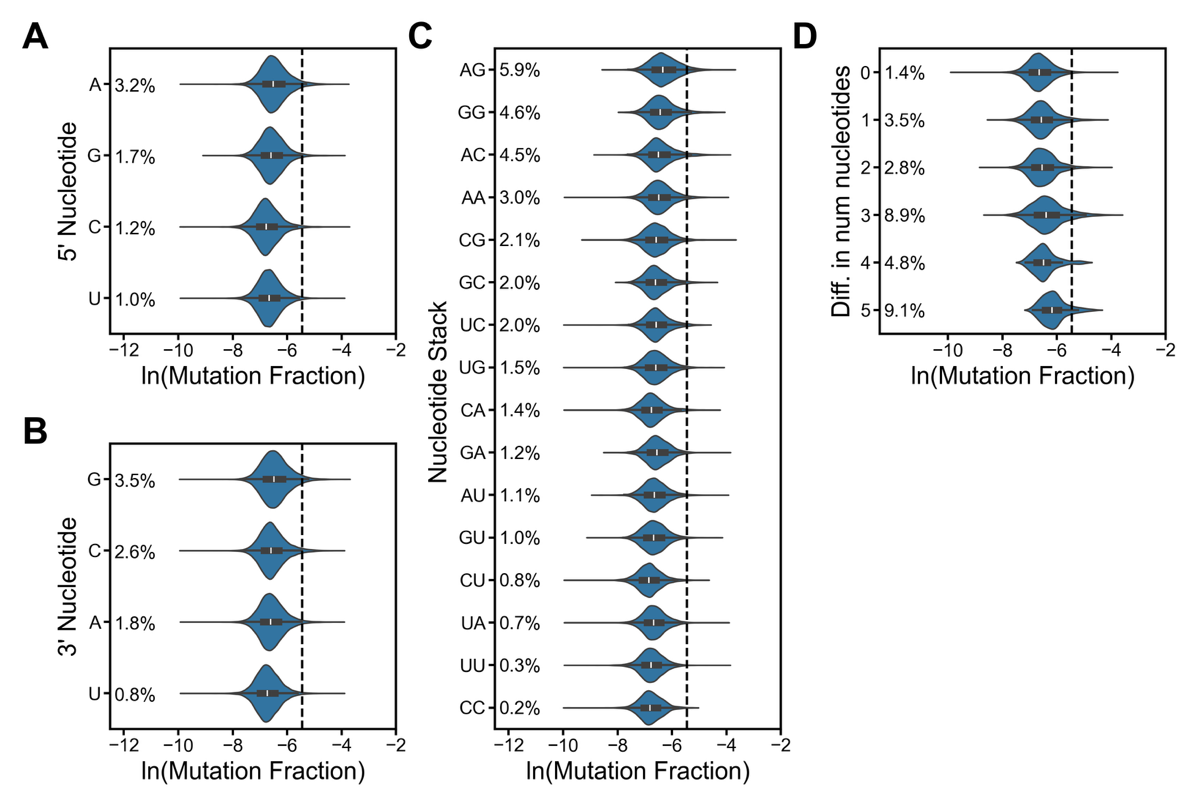


## Supplementary Figure S31: Reactive flanking base pairs for C

Remaking figures 3D-3G for cytosines. Similar trends were observed for C here as mentioned in **Figure 3D-G**.

| Motif | Nuc | Pos | Avg | SD | CV | Loop | Type | ln(DMS) |
| --- | --- | --- | --- | --- | --- | --- | --- | --- |
| CGUC_GUAG | C | 3 | 0.002 | 0.003 | 1.451 | 2,2 | WC Paired | -6.258 |
| AAGC_GAAU | C | 6 | 0.002 | 0.002 | 1.175 | 2,2 | WC Paired | -6.297 |
| UAAC_GGGA | A | 4 | 0.005 | 0.005 | 1.087 | 2,2 | Non WC Paired | -5.382 |
| ACGG_CAAU | C | 11 | 0.002 | 0.002 | 1.266 | 2,2 | WC Paired | -6.328 |
| AUCC_GCUU | A | 3 | 0.001 | 0.002 | 1.516 | 2,2 | WC Paired | -6.830 |
| GAAU_AGGC | A | 11 | 0.004 | 0.006 | 1.616 | 2,2 | WC Paired | -5.634 |
| UUGU_AAUA | A | 14 | 0.004 | 0.004 | 1.153 | 2,2 | WC Paired | -5.628 |
| CU_ACAUG | C | 3 | 0.002 | 0.003 | 1.171 | 0,3 | WC Paired | -6.052 |
| CGAAG_CAAG | C | 12 | 0.001 | 0.002 | 1.113 | 3,2 | WC Paired | -6.510 |
| UCCG_CUAA | C | 11 | 0.001 | 0.002 | 1.613 | 2,2 | WC Paired | -6.524 |
| AACU_ACCU | A | 3 | 0.003 | 0.004 | 1.371 | 2,2 | WC Paired | -5.943 |
| CACC_GCCG | C | 6 | 0.002 | 0.002 | 1.258 | 2,2 | WC Paired | -6.366 |
| UAAG_CA | C | 11 | 0.002 | 0.002 | 1.169 | 2,0 | WC Paired | -6.232 |
| CCAG_CAUG | C | 11 | 0.002 | 0.002 | 1.353 | 2,2 | WC Paired | -6.389 |
| UUGG_CGCA | C | 11 | 0.002 | 0.003 | 1.520 | 2,2 | WC Paired | -6.288 |

## Supplemental Table S3: Outliers of the coefficient of variation (CV).

This table presents outlier DMS reactivity values for nucleotides across different motifs, providing additional details about each outlier, including the coefficient of variation (CV), nucleotide type, position, loop, type, *etc.*

| Sequence | Target secondary structure | RNAstructure predicted secondary structure | DREEM – 1^st^ secondary structure for K=2 | DREEM – 2^nd^ secondary structure for K=2 | Differences |
| --- | --- | --- | --- | --- | --- |
| GGGCUUCGGCCCGUGGCAAACAAGACUGCAACUGAAGAACGUUGGCACAUCCGGACCUGCAGAAGAAAGCGAGUAGCUUUCCAGGUCAUCACGGAUGGACCAACGGAACUGCUUCAGGAACGCGCAGUCAGGAAGGCCACAAAGAAACAACAACAACAAC | ((((....))))(((((......((((((..((((((..((((((..((((((((((((.....((((((.....))))))))))))....))))))..))))))......))))))......))))))......))))).................... | 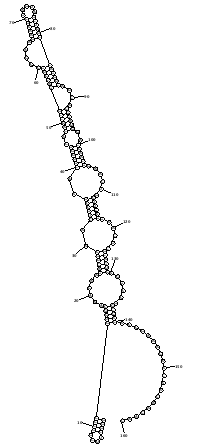 |  |  | No difference |
| GGGCUUCGGCCCGCAUCAACCAGUCAAACAAACGCUACAAGGAGGCUACCGCACCCAAGUAAUGAAACAAAGAGCUCGAGUAGAGCUCGAGACGCAUUACCGUAAAGGGUGCCCUCCUGACUGUAGCUCAUCGGACUGGAACAAAGAUGCAAAGAAACAACAACAACAAC | ((((....))))(((((..((((((........((((((((((((.....((((((..((((((.......((((((.....))))))......))))))......))))))))))))...))))))......))))))......))))).................... | 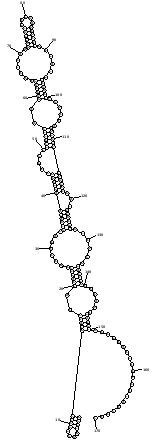 |  |  | No difference |
| GGGCUUCGGCCCUCCUCGACGACCACAACUUCUGGACCCAGUUAAGUUCCCAACCUGACAAACCCGAACACGAGUAGUGUUCAAGAGUCAGGCAAUCAGGGAACCAACUGGACAGAAGCCACUCGUGGUCCAAGAGGAAAAGAAACAACAACAACAAC | ((((....))))(((((...((((((..((((((...((((((..((((((..((((((......((((((.....))))))....))))))......)))))).)))))).))))))......))))))...))))).................... | 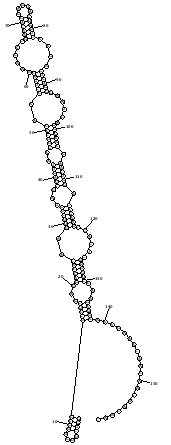 |  |  | The two clusters show a flanking base pair shift. |
| GGGCUUCGGCCCUGAUCAAACAAGACAGCAAUAAAGAGGUGAAACUGCCACCACUGUACUGAAGGACGUACAGGCGAGUAGCCUGUAAACGUCCGAGAAACAGUACUGCGGUGGCCACCUCGCUGUCAGGCGGGAUCAAAAGAAACAACAACAACAAC | ((((....))))(((((......((((((......((((((.....((((((...((((((..((((((((((((.....))))))..))))))......))))))...))))))))))))))))))......))))).................... | 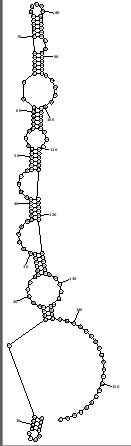 |  |  | No difference |
| GGGCUUCGGCCCGUCCCAACAUCGGAGACUUAGUCCUCAAACAGCGUUGUCACCUCUGAAGUCGACAACUCUAGUGUACGAGUAGUACACGUCGACACCAAUGCAGAGGACAACGGAAAGAGGACCCGAUGCCCUAAGGGACAAAGAAACAACAACAACAAC | ((((....))))(((((..((((((.......((((((......((((((..((((((..((((((.......((((((.....)))))))))))).......))))))))))))....))))))))))))......))))).................... | 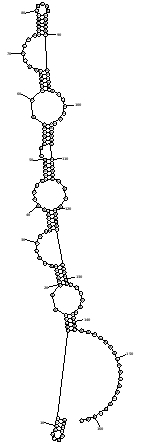 |  |  | No difference |
| GGGCUUCGGCCCGUGACCUUGCGAAAGAGACUUCGCAGACGGAGCAAACAACGACAUGGGAUGAACGUUGCGAGUAGCAACGGACAGGCAUCCCUCAUGUCGGACGAGGCUCCGACGGCGAAGCGCAAGCGUGUCACAAAGAAACAACAACAACAAC | ((((....))))(((((((((((.......((((((...((((((......((((((((((((..((((((.....))))))......))))))..))))))......))))))...))))))))))))...))))).................... | 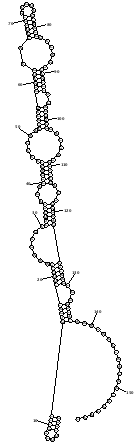 |  |  | No difference |
| GGGCUUCGGCCCAGAAGAACAAGUGAAACCAGGGUUGAAAGACCGUUGAGAGGACACAAGUAAAGAAACAAAGAGGUCGAGUAGACCUCAACAUCGGCUUUACAGCAAGGGUGUCCCAUCAACGCAACCCACAGCACUUGAAGCCAGCUUCUAAAGAAACAACAACAACAAC | ((((....))))(((((..((((((......((((((......((((((..((((((..((((((.......((((((.....))))))........)))))).......))))))..))))))))))))....)))))).......))))).................... | 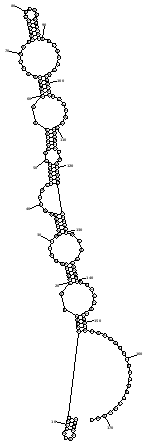 |  |  | No difference |
| GGGCUUCGGCCCAUGUGAAACAAAGUAACCAAGUAUACCCGUUUACCGGUAGAGAAACGGCUAGACUUGUUUCGAGUAGAAACACUAGCCACUACUGCUCUACGUAAACGUAUACAGGAAGCGGUUACAAGACGAGCACAUAAAGAAACAACAACAACAAC | ((((....))))(((((.......((((((..((((((..((((((..((((((....((((((...((((((.....)))))))))))).......)))))))))))))))))).......))))))........))))).................... | 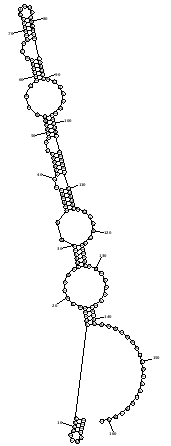 |  |  | The two clusters show a flanking base pair shift. |
| GGGCUUCGGCCCAUACCGACUACGCAGUUAUCAAACCAGUUACGAAACAAGCACACAUUCUACUUAAGCUCGAGUAGAGCUUCAAAGUAGUUAGUGUGCGGCGCGCGUAACAGCCGAUAACCAAGUAGUCCUCGGUAUAAAGAAACAACAACAACAAC | ((((....))))(((((((((((...((((((......((((((......((((((...((((((((((((.....))))))..))))))...))))))......))))))....))))))...))))))...))))).................... | 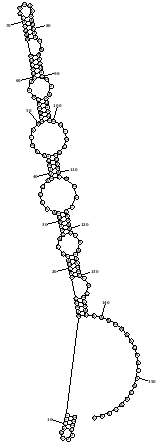 |  |  | The two clusters show flanking base pair shifts. |
| GGGCUUCGGCCCUGAGCAAACGAAACCAACUUAUGAGAGCGAGAUAUCACAAGCUCGUCGCACGCGCUACAAACAAACGAAACCGAGUAGGUUUCAACAGAUAGUAGCGGACGAGGAUAUCCAUAAGCUACCCGGUUUCAGAACAAGCUCAAAAGAAACAACAACAACAAC | ((((....))))(((((....((((((..((((((.......((((((.....((((((.....((((((........((((((.....))))))........))))))))))))))))))))))))......)))))).......))))).................... | 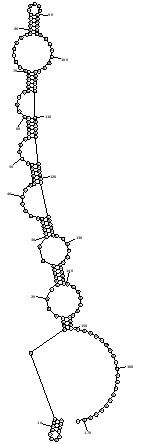 |  |  | Show significant secondary structure rearrangement in the two clusters (observed in two of the motifs in this construct) |

## Supplementary Table S5: DREEM clustering on preliminary constructs

Table of DREEM clustering results on preliminary construct design.

| Category | Percent below 2 Å | Count |
| --- | --- | --- |
| A in A-A | 23.29 | 992 |
| A in A-C | 62.43 | 668 |
| A in A-G | 69.25 | 374 |
| C in C-A | 51.66 | 664 |
| C in C-C | 12.06 | 506 |
| C in C-U | 0.00 | 521 |

## Supplemental Table S6: Residues under 2 Å for solvent accessible surface area.

Table shows the number of residues and their corresponding percentages that are under 2 Å for solvent accessible surface area (SASA) for each base pair mismatch category.
